# Amorphous BiSn_x_O_y_ for Efficient CO_2_ Electroreduction to Formate via In Situ Doping

**DOI:** 10.1002/advs.202522395

**Published:** 2025-12-30

**Authors:** Zhenjie Cheng, Junnan Song, Lijia Liu, Chenglong Qiu, Lu Wang, Jiacheng Wang

**Affiliations:** ^1^ Zhejiang Key Laboratory for Island Green Energy and New Materials School of Materials Science and Engineering Taizhou University Taizhou P. R. China; ^2^ Department of Chemistry Western University London Ontario Canada; ^3^ Key Laboratory of Green and Precise Synthetic Chemistry and Applications Ministry of Education Anhui Provincial Key Laboratory of Synthetic Chemistry and Applications College of Chemistry and Materials Science Huaibei Normal University Huaibei P. R. China

**Keywords:** amorphous materials, CO_2_ reduction, formate, metamorphosis, theoretical calculation

## Abstract

The practical implementation of electrochemical CO_2_ reduction to formate has been limited by persistent issues concerning product selectivity, operational current density, and long‐term stability. To address these challenges, we developed an amorphous BiSn_x_O_y_ precatalyst capable of overcoming conventional activity‐stability compromises, enabling efficient and durable formate production at industrially relevant current densities. The amorphous structure exhibits significantly reduced oxygen vacancy formation energy compared to its crystalline counterpart, facilitating rapid structural transformation under electrocatalytic conditions. Remarkably, this catalyst demonstrates exceptional performance, achieving a Faradaic efficiency (FE_Formate_) of 95.6% at 800 mA cm^−2^ in a flow cell, maintaining stable operation at 500 mA cm^−2^ in a membrane electrode assembly (MEA) electrolyzers, and delivering an FE of 92.3% for over 160 h at 200 mA cm^−2^. We further validated the practical applicability by integrating the catalyst into a solar‐powered MEA system for sustainable formate generation. Through comprehensive in situ spectroscopic characterization and density functional theory (DFT) calculations incorporating crystal orbital Hamilton population (COHP) analysis, we elucidate that Sn incorporation tailors the electronic configuration of Bi sites, optimizing the binding of the crucial *OCHO intermediate for selective formate formation. This work establishes a dynamic catalyst paradigm that transcends classical activity‐stability tradeoffs, charting an atom‐efficient pathway for industrial CO_2_ valorization using renewable energy.

## Graphical abstract

The amorphous BiSn_x_O_y_ catalyst is electroreduced to Sn‐doped Bi during the electrochemical process. The Sn‐modulated Bi exhibits a favorable intermediate adsorption potential, leading to optimal catalytic efficiency. Remarkably, this catalyst demonstrates exceptional performance, achieving a Faradaic efficiency (FE_formate_) of 95.6% at 800 mA cm^−2^ in a flow cell, maintaining stable operation at 500 mA cm^−2^ in a membrane electrode assembly (MEA) electrolyzer, and sustaining an FE of 92.3% for over 160 h at 200 mA cm^−2^.

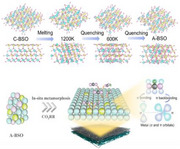

## 1 Introduction

Electrochemical conversion of CO_2—_the primary greenhouse gas emitted from anthropogenic activities—into carbon‐neutral fuels and value‐added chemicals using renewable electricity has emerged as a promising strategy for sustainable carbon utilization [1, 2, 3, 4]. To date, a broad spectrum of CO_2_ reduction reaction (CO_2_RR) products has been reported, including CO, CH_4_, HCOO^−^/HCOOH, and various C_2+_ hydrocarbons and oxygenates [5, 6, 7]. Although C_2+_ products generally have a larger global market and higher commercial value than C_1_ chemicals, their relatively low energy efficiency (EE) and selectivity limit their economic feasibility [8, 9]. Among various CO_2_ reduction products, formic acid and formate have garnered significant interest due to their widespread applications in industrial synthesis and formic acid fuel cells, as well as their relatively low activation potential, requiring only a two‐electron transfer process. Therefore, developing highly efficient catalysts with excellent activity, superior selectivity, and long‐term stability remains both crucial and challenging for the large‐scale practical application of formate production [10, 11, 12].

Several p‐block metals, such as In, Bi, Pb, and Sn, have been extensively explored as promising electrocatalysts for CO_2_ reduction to formate. Their high selectivity originates from their favorable affinity for stabilizing the key oxygen‐bound intermediate (*OCHO), which steers the reaction pathway toward formate instead of CO or other products [13, 14]. Bi‐based materials have emerged as promising candidates among various electrocatalysts due to their cost‐effectiveness, environmental friendliness, high selectivity, and excellent catalytic activity. In pursuit of higher EE, delicate catalyst design has been carried out in past years toward an evenly improved reaction kinetics and FE of CO_2_‐to‐HCOO^−^ conversion. Strategies such as the fabrication of Bi nanosheets or nanotubes [15, 16], exposing specific crystal facets [17, 18], defect or grain boundary engineering [19], and alloying with a second metal have been developed to enhance the performance of Bi‐based catalysts [20]. Among these approaches, heteroatom doping is particularly attractive, as it effectively modulates the electron density around the host atoms, thereby optimizing their adsorption and desorption properties for key reaction intermediates, ultimately improving catalytic efficiency and selectivity [21, 22, 23, 24].

Amorphous nanomaterials possess a long‐range disordered structure, with surfaces rich in highly unsaturated atomic sites and dangling bonds, which provide abundant active sites. Moreover, the presence of highly flexible local structures in the amorphous phase can facilitate charge transfer between active sites and key intermediates, thereby improving overall catalytic performance [25, 26, 27]. Although the application of amorphous nanomaterials in electrocatalysis has attracted widespread attention, their exploration as catalysts in the CO_2_RR is still in its early stages. While several studies have investigated the use of amorphous catalysts in CO_2_RR, the research focus has largely remained on performance evaluation, with limited further investigation into the catalytic mechanisms and structural evolution [28]. Critical questions regarding their CO_2_RR performance—such as structural stability under high current densities and the underlying catalytic mechanisms—remain unanswered and warrant systematic investigation.

Herein, we successfully synthesized amorphous bismuth‐tin oxide (BiSn_x_O_y_, denoted as A‐BSO) pre‐catalysts toward highly efficient and stable CO_2_RR for formate formation with high FEs. In comparison to crystalline Bi_2_Sn_2_O_7_ (denoted as C‐BSO), A‐BSO undergoes electrochemical reconstruction under CO_2_RR working potentials, leading to in situ formation of a Sn‐doped metallic Bi catalyst (denoted as Sn@Bi). The A‐BSO nanoparticles exhibit exceptional CO_2_RR activity for selective formate production across a wide potential window. In a flow cell configuration incorporating a gas diffusion electrode (GDE), A‐BSO demonstrates exceptional performance, delivering an ultrahigh current density of 800 mA cm^−2^ (∼1.3 V vs. RHE) while maintaining 95.6% FE for formate production. Similarly, in the MEA configuration, the catalyst demonstrates comparable performance, sustaining a current density of 270 mA cm^−2^ at 3.6 V full‐cell voltage while maintaining 94.3% FE for formate production. Notably, the system exhibits exceptional operational stability, showing no significant performance decay during a 160‐h continuous test at 200 mA cm^−2^. DFT calculations reveal that Sn doping into the Bi matrix fine‐tunes the electronic structure of surface Bi sites and modulates the adsorption strength of the *OCHO intermediate. Further analysis indicates electron transfer from Bi to Sn, which facilitates the adsorption of electron‐rich *OCHO on electron‐deficient Bi sites. To enable renewable energy‐driven CO_2_ reduction to formate, we utilized a monocrystalline silicon solar panel to power the MEA electrolyzer and successfully achieved the conversion with an overall energy efficiency of approximately 5%.

## 2 Results and Discussion

**FIGURE 1:**
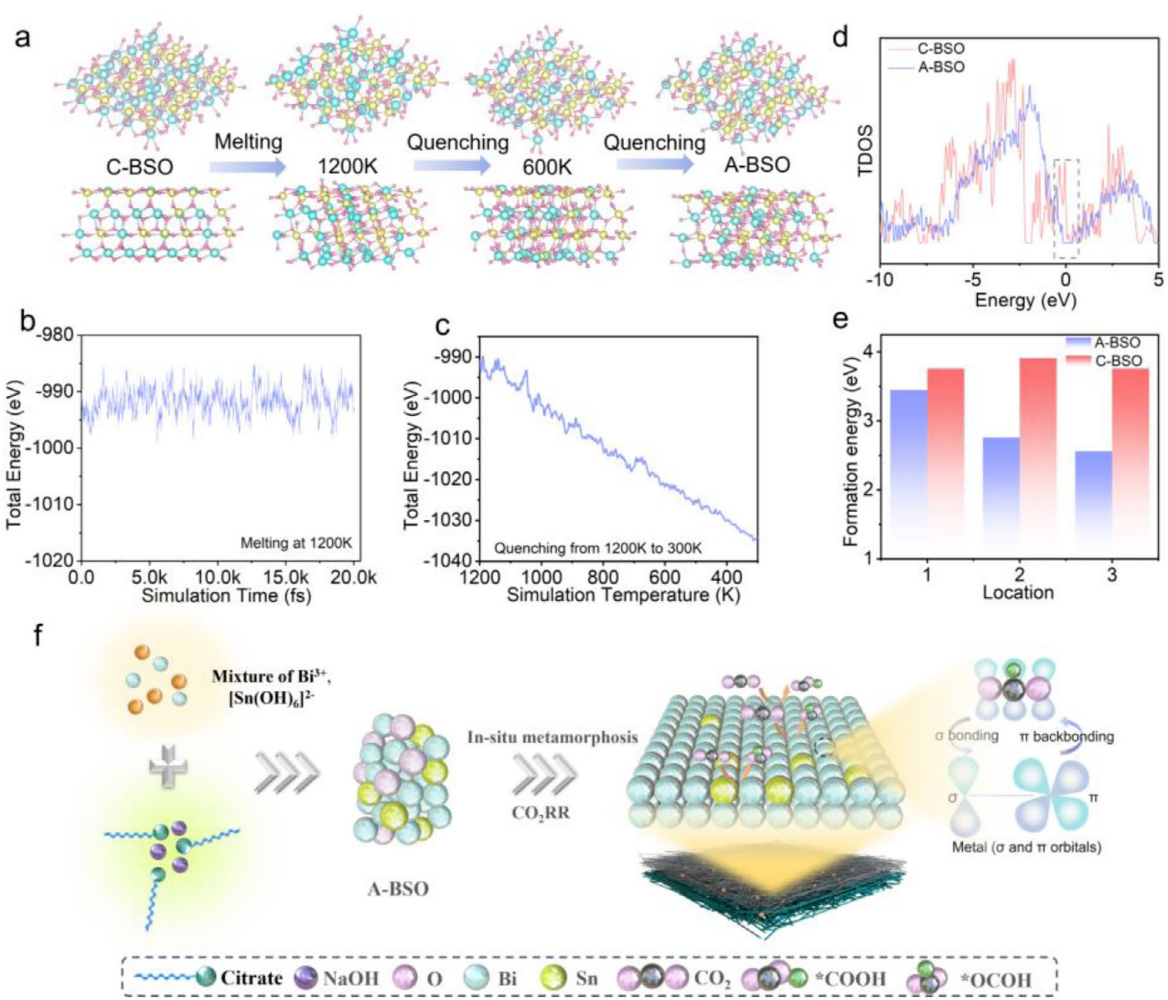
(a) The heating‐quenching process of C‐BSO transitioning to the amorphous state (A‐BSO). (b) The system energy changes and the corresponding time curve during the heating process. (c) The energy variation curve during the quenching process. (d) DOS diagrams of C‐BSO and A‐BSO. (e) Formation energy of oxygen vacancies at different sites (1, 2, 3) in C‐BSO and A‐BSO. (f) Schematic of in situ metamorphosis engineering of A‐BSO pre‐catalysts for CO_2_RR to formate formation.

The structural transition process from C‐BSO to A‐BSO was simulated using molecular dynamics, as shown in Figure 1a. The increasing atomic disorder significantly disrupts the crystalline symmetry of the material. When the crystal is in a high‐temperature state, the increased kinetic energy of atoms leads to higher internal energy (Figure 1b). During the simulation of quenching the high‐temperature melt to room temperature, the total energy of the system gradually decreases (Figure 1c). Density of states (DOS) calculations reveal that the amorphous structure exhibits localized electronic states near the Fermi level, which enhances electron‐electron repulsion and consequently leads to higher Gibbs free energy in the amorphous state (Figure 1d) [26]. This energy difference between the two structures results in distinct capabilities to maintain structural stability during electrochemical reduction processes. Comparative analysis of oxygen vacancy formation energies at different sites reveals that A‐BSO consistently exhibits lower formation energies across various sites compared to C‐BSO (Figure 1e). This indicates that A‐BSO is more prone to oxygen loss during electrochemical reduction processes. Figure 1f clearly illustrates the phase evolution of A‐BSO during electrochemical reduction, demonstrating its tendency to be reduced into Sn‐doped Bi metal. The derived Sn@Bi catalyst exhibits both strong *OCHO intermediate adsorption energy and weak *H adsorption energy, creating an optimal electronic environment that significantly improves the Faradaic efficiency for formate production in the CO_2_RR process.

**FIGURE 2:**
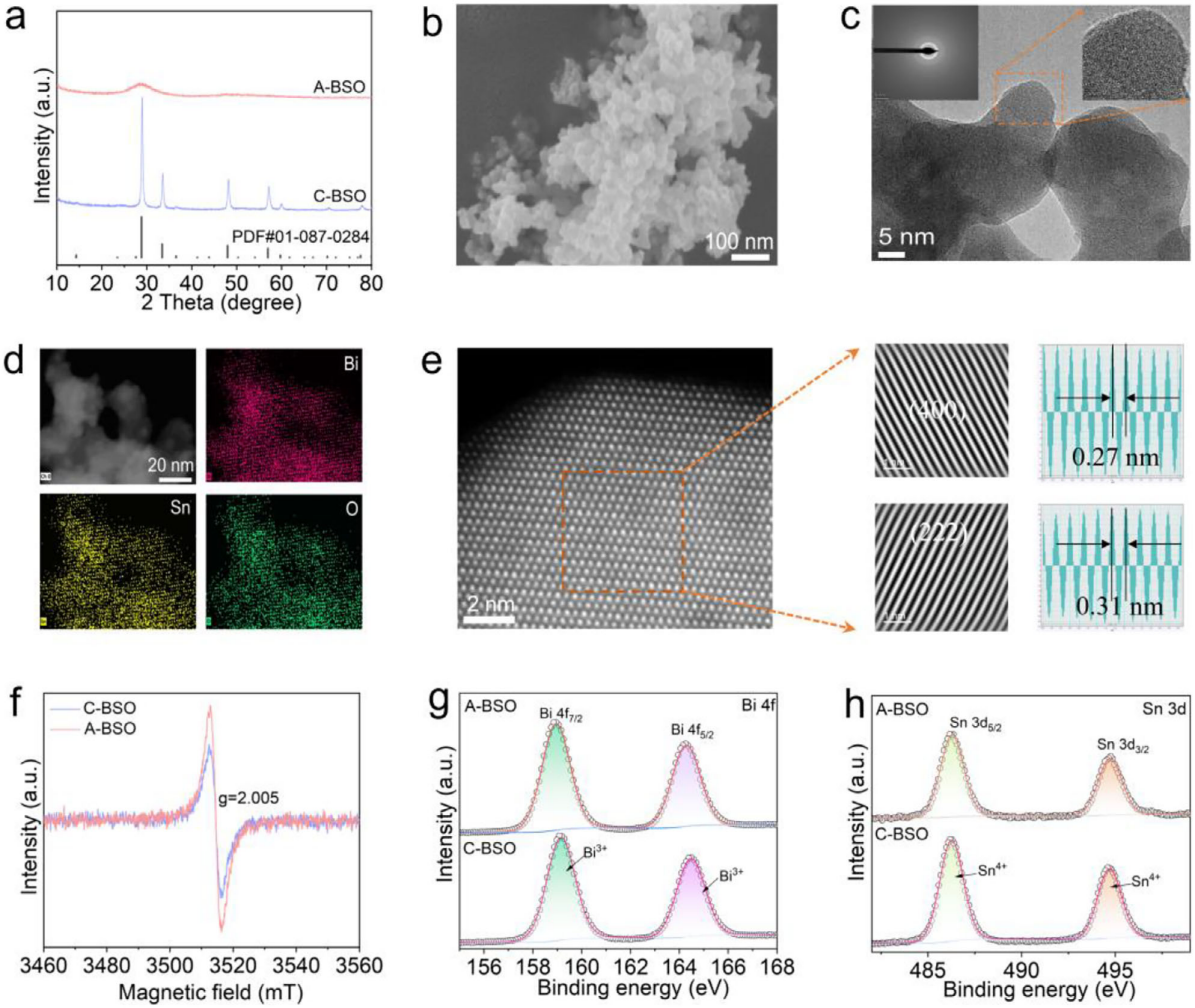
Structural characterization of C‐BSO and A‐BSO. (a) XRD patterns of C‐BSO and A‐BSO. (b) SEM image of A‐BSO. (c) HR‐TEM image of A‐BSO, with the corresponding SAED pattern shown in the upper left inset. (d) Scanning TEM (STEM) image and corresponding EDS elemental mappings of Bi, Sn, and O for the A‐BSO sample. (e) Atomic‐resolution HAADF‐STEM image and the corresponding lattice fringes obtained by inverse FFT from the selected region, indexed to the (222) and (400) planes of C‐BSO. (f) EPR spectra of A‐BSO and C‐BSO. (g) Bi 4f and (h) Sn 3d XPS spectra of A‐BSO and C‐BSO, respectively.

Experimental characterizations were systematically performed to validate the above theoretical results. As shown in Figure 2a, the diffraction peaks of C‐BSO exhibit angles and intensities that align well with the standard reference for Bi_2_Sn_2_O_7_ (PDF#01‐087‐0284). In contrast, the diffraction pattern of A‐BSO is characterized by a prominent amorphous diffuse reflection peak at 28°, indicating its non‐crystalline nature. Figure 2b reveals that A‐BSO exhibits a morphology of nanoparticle aggregates, which is remarkably similar to that of C‐BSO (Figure S2). High‐resolution Transmission Electron Microscopy (HR‐TEM) provides atomic‐scale resolution of structural characteristics of A‐BSO and C‐BSO. Figure 2c presents the HR‐TEM image of as‐synthesized A‐BSO and its corresponding selected‐area electron diffraction (SAED) pattern. The worm‐like lattice fringes, combined with the diffuse halo in the SAED pattern (inset), collectively confirm the amorphous nature of the material, as evidenced by the absence of distinct diffraction spots or rings characteristic of crystalline structures [29, 30, 31]. Energy‐dispersive spectroscopy (EDS) elemental mapping confirms the uniform distribution of Bi and Sn at the atomic scale (Figure 2d). In contrast, atomic‐resolution HAADF‐STEM imaging of C‐BSO, together with lattice fringes extracted by inverse FFT from the selected region, clearly identifies the (222) and (400) planes (Figure 2e). Elemental mapping further confirms the presence and uniform distribution of Bi and Sn in C‐BSO (Figure S3).

Amorphous metal oxides typically exhibit higher concentrations of bulk‐phase defects and oxygen vacancies relative to their crystalline analogues. Electron paramagnetic resonance (EPR) was employed to characterize both materials (Figure 2f). In contrast to C‐BSO, A‐BSO displays a pronounced EPR signal at g = 2.005, suggesting a high density of unsaturated atomic sites within its bulk phase, which contributes to the formation of numerous defects and oxygen vacancies. The chemical surface composition and electronic structure of A‐BSO and C‐BSO were further analyzed using X‐ray photoelectron spectroscopy (XPS) (Figure S4). Figure 2g,h display the XPS survey spectra along with their corresponding deconvolution results for the Bi 4f and Sn 3d core‐level regions, respectively. Figure 2g reveals characteristic Bi 4f doublets (4f_7/2_ and 4f_5/2_) in both A‐BSO and C‐BSO, with binding energies consistent with Bi^3+^ oxidation state. Correspondingly, Figure 2h demonstrates Sn 3d peaks indicative of Sn^4+^, confirming that both metals maintain their typical oxide coordination environments (M─O, M = Bi, Sn) despite structural differences between amorphous and crystalline phases.

**FIGURE 3:**
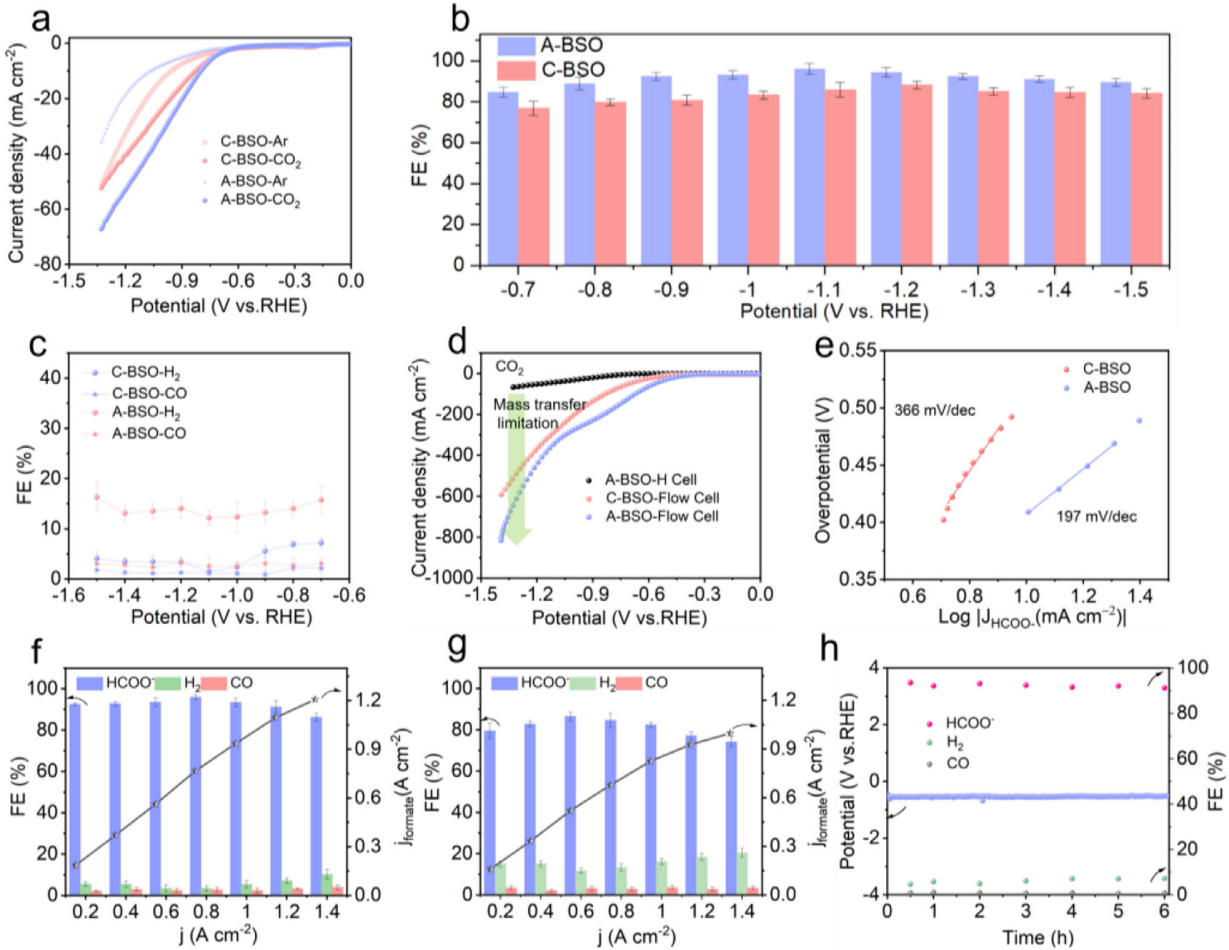
CO_2_ electroreduction performance. (a) LSV curves of C‐BSO and A‐BSO in an H‐type cell. (b and c) FEs of HCOO^−^ (b), and H_2_ and CO (c). (d) LSV curves of A‐BSO and C‐BSO in a flow cell, with A‐BSO performance in an H‐cell shown for comparison. (e) Tafel plots for CO_2_ electroreduction to HCOO^−^ on A‐BSO and C‐BSO. (f and g) FEs and partial current densities of A‐BSO (f), and C‐BSO (g) at different current densities from 0.2 to 1.4 A cm^−2^ in 1.0 M KOH. (h) Stability test of A‐BSO at a constant current density of −200 mA cm^−2^ in 1 M KOH using a flow cell.

To assess the CO_2_ electroreduction activity of A‐BSO and C‐BSO, catalytic reactions were carried out in H‐type cells, with linear sweep voltammetry (LSV) measurements performed in 0.5 M KHCO_3_ at a scan rate of 10 mV s^−1^. As depicted in Figure 3a, the current densities for both catalysts under a CO_2_ atmosphere are substantially higher than those under an Ar atmosphere, indicating their intrinsic activity for CO_2_ reduction [32, 33]. Notably, at the same overpotential, A‐BSO generates a significantly higher reduction current for CO_2_ compared to C‐BSO, suggesting that A‐BSO may exhibit superior catalytic performance. To identify the reduction products, electrolysis at a range of selected potentials ranging from −0.7 to −1.5 V (VS. RHE) was performed. Gas‐phase products were analyzed using gas chromatography (GC) and differential electrochemical mass spectrometry (DEMS) (Figures S5 and S6), while liquid‐phase products were characterized by nuclear magnetic resonance (^1^H NMR) spectroscopy (Figure S7). The FEs summarized at various potentials reveal that formate is the predominant product among all CO_2_ reduction products, highlighting the exceptional selectivity of both catalysts. Compared to the C‐BSO catalyst, the A‐BSO exhibits superior formate selectivity in CO_2_RR. The formate FE at the A‐BSO electrode reaches up to 96.2% at −1.1 V and remains above 90% over a wide potential range (Figure 3b). In sharp contrast, the control experiments using the C‐BSO electrode achieve a maximum FE of only 88.1% at −1.2 V. Gas‐phase product analysis revealed only H_2_ and CO for both catalysts. While their CO FEs were comparable, C‐BSO exhibited significantly enhanced H_2_ production due to its superior HER activity (Figure 3c).

The electrochemically active surface area (ECSA) of both catalysts was measured before and after electrolysis to investigate changes in the active surface area during the CO_2_RR process (Figure S8) [34, 35]. Based on the double‐layer capacitance (C_dl_) per unit area, it is evident that the active surface area of A‐BSO was slightly larger than that of C‐BSO before electrolysis. After the electrolysis process, the active surface area of A‐BSO experienced a notable increase. Nevertheless, this expansion in surface area alone does not fully account for the observed improvements in both activity and selectivity. Given that the Bi content in A‐BSO is significantly higher than that in C‐BSO, and considering that Bi inherently possesses a greater formate conversion rate compared to Sn (Table S1), it is reasonable to hypothesize that this difference contributes to the enhanced formate selectivity observed in A‐BSO. However, even when compared to nanoscale Bi_2_O_3_, A‐BSO still exhibits superior formate selectivity (Figure S9). This suggests that the higher Bi content alone cannot entirely account for the increased formate FE in A‐BSO, indicating that other factors, such as structural or electronic properties, may also play a critical role in its performance [36, 37]. To overcome the mass diffusion limitations caused by the low solubility of CO_2_ in water, the CO_2_ electroreduction performance of both catalysts was evaluated using a GDE (Figure S10). As illustrated in Figure 3d, following the enhancement of CO_2_ mass transfer, both catalysts exhibited a substantial increase in current densities at higher polarization voltages. Specifically, C‐BSO achieved a current density of approximately 800 mA cm^−2^ at a polarization voltage of −1.3 V, significantly surpassing the 68 mA cm^−2^ recorded in the H‐type cell. Moreover, the onset potential of around −0.4 V was notably lower than that observed in the H‐type cell (−0.6 V), likely due to the reduced energy barrier for the CO_2_RR and the suppression of the HER in the alkaline electrolyte.

Tafel analysis was performed on different electrocatalysts to investigate their reaction kinetics. As illustrated in Figure 3e, the Tafel slope of A‐BSO (197 mV dec^−1^) was significantly lower than that of C‐BSO (366 mV dec^−1^), suggesting that the A‐BSO nanostructure exhibits faster reaction. Although CO_2_ exhibits faster reaction kinetics on the A‐BSO surface, its selectivity in the flow cell needs further investigation. In the flow cell, the main products of CO_2_ reduction for both catalysts are H_2_, CO, and formate. For A‐BSO, the FE for formate remains above 90% across a wide range of current densities, reaching as high as 95.6% at 0.8 A cm^−2^ (Figure 3f). The combination of high total current density and high FE enables an industrial ampere‐level partial current density for formate production. In sharp contrast, the C‐BSO catalyst exhibits significant HER activity, leading to substantially higher H_2_ production compared to A‐BSO. As a result, the FE for formate remains around 80% across the tested current density range, which is clearly suboptimal (Figure 3g). The stability of A‐BSO is carried out through a long‐term chronopotentiometry at −200 mA cm^−2^. As shown in Figure 3h, the electrode potential remains relatively stable with increasing electrolysis time, indicating that the catalyst surface retains excellent hydrophobicity. Furthermore, the FE for formate consistently exceeds 90% over prolonged operation, demonstrating the remarkable stability of A‐BSO. In stark contrast, C‐BSO demonstrates markedly inferior stability, exhibiting continuous potential polarization during electrolysis while maintaining substantially lower formate FE (Figure S12). This pronounced performance disparity highlights A‐BSO's exceptional operational stability and catalytic durability under identical test conditions.

**FIGURE 4:**
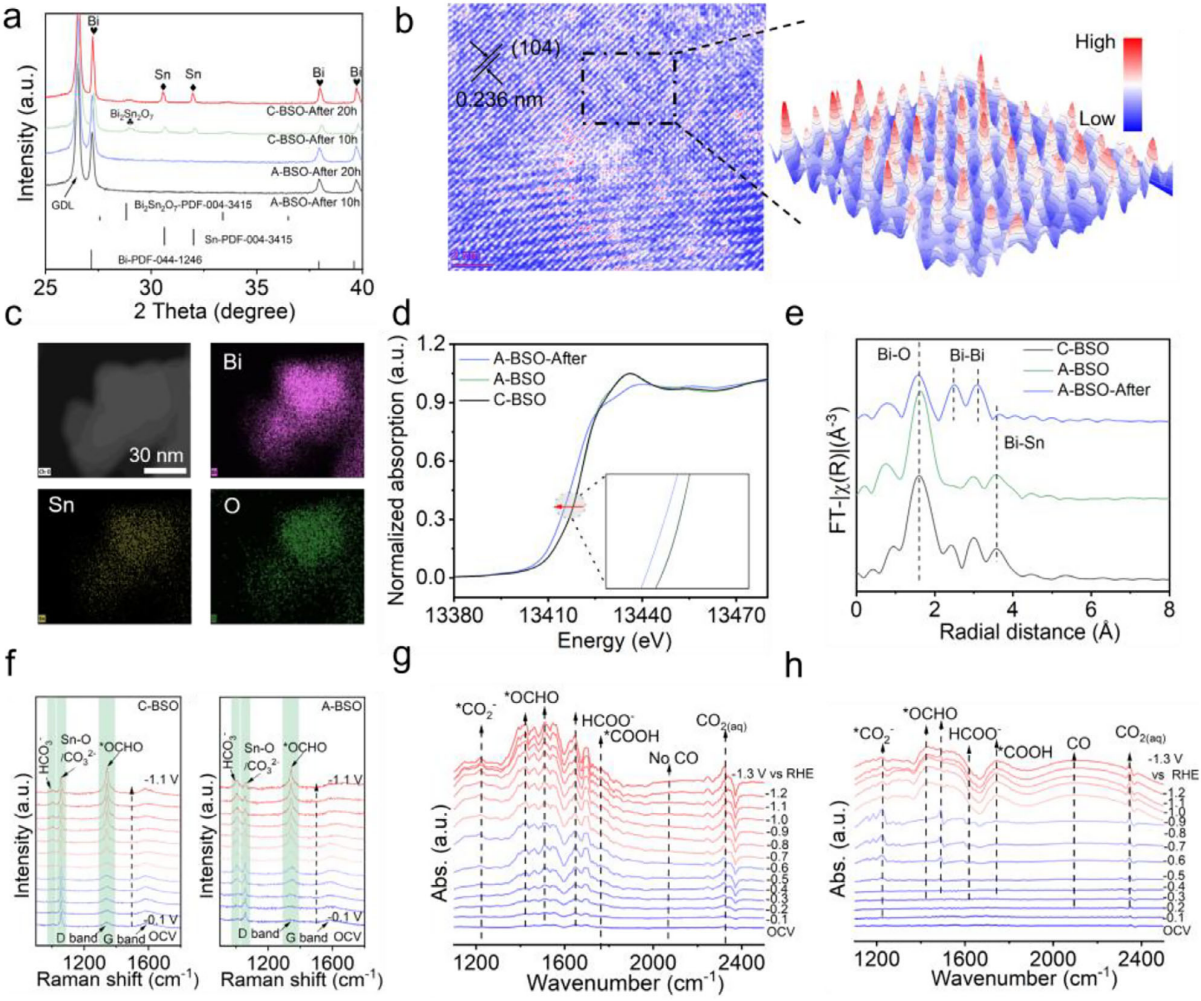
Structural characterization of A‐BSO and C‐BSO electrocatalysts toward CO_2_RR. (a) XRD patterns of A‐BSO and C‐BSO after electrochemical reduction at 30 mA cm^−2^ for different durations. (b) HR‐TEM image of electrochemically reduced A‐BSO with atomic‐resolution 3D topographic mapping from selected‐area analysis. (c) STEM image and corresponding EDS elemental mappings of reduced A‐BSO. (d) Normalized XANES and (e) FTk^3^χ(k) EXAFS of Bi L_3_‐edge EXAFS signals for different samples. (f) In situ Raman spectra of C‐BSO and A‐BSO under CO_2_RR at various potentials in 0.5 M KHCO_3_ electrolyte. In situ SEIRAS spectra of (g) A‐BSO and (h) C‐BSO at various potentials in CO_2_‐saturated 0.5 M KHCO_3_ electrolyte.

To further elucidate the reasons behind the superior CO_2_ reduction performance of A‐BSO compared to C‐BSO, additional characterization tests are necessary to provide supporting evidence. Substantial experimental evidence has established that structural reconstruction of metal‐based compounds constitutes a ubiquitous but complex phenomenon during CO_2_RR. Precise identification of the reconstructed catalytic phases is crucial for elucidating the fundamental structure‐activity relationships and operative reaction mechanisms. By comparing the XRD patterns after different electrolysis durations, it can be observed that both A‐BSO and C‐BSO exhibit diffraction peaks corresponding to Bi. For the C‐BSO sample, diffraction peaks from both the pristine C‐BSO and newly formed Sn are still clearly detectable (Figure 4a). Local EDS analysis further indicates that in the crystalline regions resulting from partial decomposition of C‐BSO (Figure S13b,c), distinct Sn, Bi, and C‐BSO‐derived products are present, with the Sn content in some regions being significantly higher than in the pristine C‐BSO material (Figure S13a). On the other hand, no Sn element was detected in the Bi regions generated from the decomposition of C‐BSO (Figure S13c). In contrast, the Bi produced by the electrochemical reconstruction of A‐BSO contains obvious Sn elements, which are likely attributable to a Sn doping effect (Figure S13d).

The transition from amorphous to crystalline phase in A‐BSO is unequivocally demonstrated by HR‐TEM, where the emergence of (104) lattice planes coexists with defect‐rich regions (Figure 4b). This heterogeneous crystallization pattern indicates nucleation‐dominated phase transformation. Furthermore, three‐dimensional atomic imaging analysis demonstrates significant variations in atomic imaging intensity, which can be attributed to the presence of heterogeneous atomic doping [38, 39]. This doping effect is also responsible for the observed distortions in the crystal planes. As shown in Figure 4c, the reconstructed A‐BSO remains an aggregate of nanoparticles, with EDS confirming that some Sn is still retained in the structure. To reveal the valence state and coordination environment of Bi in A‐BSO before and after electrochemical reconstruction, X‐ray absorption spectroscopy (XAS) analysis was conducted. As shown in Figure 4d, the Bi L_3_‐edge X‐ray absorption near‐edge structure (XANES) spectra reveal that pristine A‐BSO and C‐BSO share nearly identical absorption edge energies, confirming equivalent initial Bi oxidation states. After reconstruction, A‐BSO exhibits a pronounced shift toward lower energy, indicating Bi reduction to a lower oxidation state, consistent with XPS results (Figure S14). The coordination structure was ascertained by Fourier transforms extended X‐ray absorption fine structure (FT‐EXAFS) (Figure S15). As shown in Figure 4e, the FT‐EXAFS spectra display a distinct Bi‐O coordination at around 1.8 Å for the pristine A‐BSO and C‐BSO [20, 40, 41]. After electrochemical reduction, the reconstructed A‐BSO exhibits intensified Bi─Bi bonding and weak Bi─Sn bonds. Based on the above experiments, it can be concluded that after reconstruction, A‐BSO is reduced to a crystalline structure of Bi with Sn doping.

To investigate the key intermediates and the reaction microenvironment on the catalyst surface during CO_2_RR, in situ electrochemical Raman spectroscopy analysis was conducted (Figure S16). Initial spectroscopic analysis at OCV reveals: (i) a distinct vibrational mode at 1065 cm^−1^ corresponding to Sn─O stretching [42, 43], and (ii) well‐defined G band (1580 ± 2 cm^−1^) and D band (1350 ± 3 cm^−1^) characteristic of graphitic carbon domains, indicating the coexistence of metal oxide and carbon support components (GDE) (Figure S17) [44, 45]. Raman spectral analysis of C‐BSO reveals voltage‐dependent evolution of key intermediates: While the Sn─O vibrational mode at 1065 cm^−1^ remains invariant with applied potential, two reactive species demonstrate dynamic behavior—the HCO_3_^−^ symmetric stretching at 1016 cm^−1^ and the *OCHO deformation mode at 1350 cm^−1^, suggesting potential‐dependent intermediate stabilization. The bicarbonate signal originates from the reaction between locally generated OH− and dissolved CO_2_ at the electrode‐electrolyte interface, whereas the *OCHO vibrational feature stems from the chemisorption of this key reaction intermediate on bismuth active sites [3, 46, 47, 48]. For A‐BSO, the peak at 1065 cm^−1^ gradually weakens throughout the electrolysis process, primarily due to structural reconstruction (Figure 4f). Moreover, compared to C‐BSO, the peaks corresponding to *OCHO and HCO_3_ appear at a lower polarization voltage, indicating that CO_2_ is more readily reduced to HCOO^−^ on the surface of A‐BSO.

In situ SEIRAS, as the most sensitive technique for capturing reactive intermediates during CO_2_RR, was employed to investigate C‐BSO and A‐BSO in a CO_2_‐saturated 0.5 M KHCO_3_ electrolyte under applied potentials ranging from −0.1 to −1.3 V vs. RHE [49, 50]. The potential‐dependent spectra reveal a continuous increase in the peak intensity at 1270 cm^−1^, corresponding to the *CO_2_^−^ intermediate, demonstrating its persistent formation during electrolysis (Figure 4g) [51, 52]. Notably, two diagnostic peaks at 1420 cm^−1^ (symmetric O‐C‐O stretch) and 1530 cm^−1^ (asymmetric O‐C‐O stretch) are observed, both characteristic of the *OCHO intermediate—the critical precursor for formate production [53, 54]. While *OCHO is detected on both catalysts, C‐BSO exhibits a significantly stronger signal at 1750 cm^−1^, assigned to *COOH, an intermediate that favors CO formation (Figure 4h). This mechanistic distinction aligns with the concurrent detection of a prominent CO stretching vibration (∼2040 cm^−1^) on C‐BSO, underscoring its divergent reaction pathway compared to formate‐selective behavior for A‐BSO [55, 56].

**FIGURE 5:**
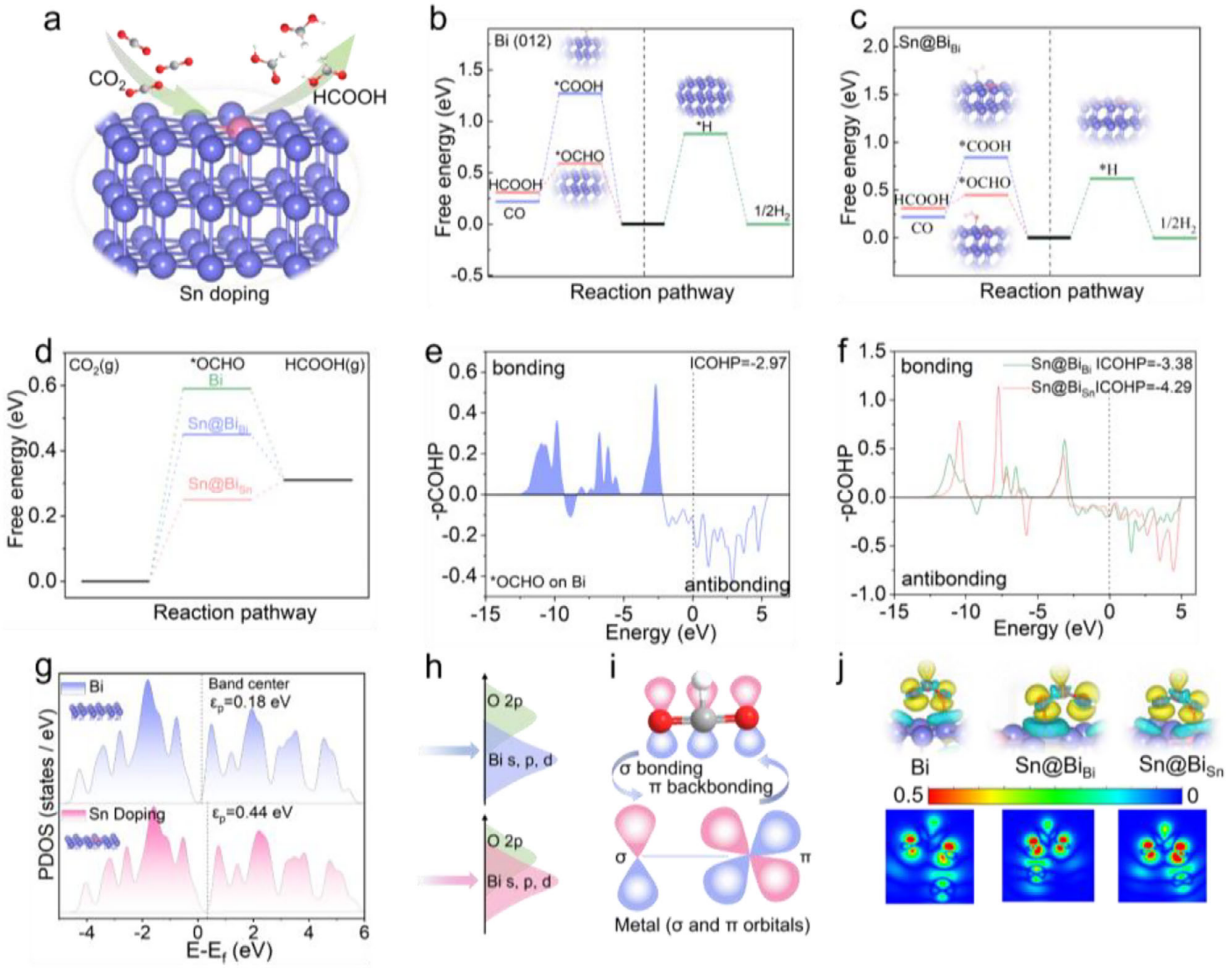
DFT Calculations. (a) Schematic representation of CO_2_RR on the surface of Sn@Bi crystal model. (b) Free‐energy diagrams for HCOOH, CO, and H_2_ formation on Bi (012) plane. (c) Free‐energy diagrams for HCOOH, CO, and H_2_ formation on Sn@Bi_Bi_ (012) plane. (d) Gibbs free energy diagram for CO_2_RR to HCOOH at different active sites. (e) COHP and the corresponding ICOHP values for Bi─O bonding of *OCHO adsorption on Bi surfaces. (f) COHP and the corresponding ICOHP values for Bi─O and Sn─O bonding of *OCHO adsorption on Sn@Bi surfaces. (g) PDOS of Bi 6p orbitals of Bi and Sn@Bi and weighted band center for two models without adsorbate. (h) Schematic illustration for PDOS overlapping areas of Bi s, p, and d orbitals on Bi and Sn@Bi interface with O 2p orbitals of *OCHO. (i) Scheme of σ bonding and π backbonding between *OCHO and Bi. (j) Calculated volume slices of calculated charge densities and corresponding optimized configurations for three models with *OCHO adsorbate.

]The structure‐activity relationship of the Sn@Bi catalyst derived from in situ reconstruction of A‐BSO was systematically investigated through DFT calculations, with particular emphasis on correlating electronic properties and atomic configurations with catalytic performance (Figure S18). Based on XRD characterization confirming the predominant exposure of Bi (012) crystallographic planes in Sn@Bi, all computational models were constructed using the Sn@Bi (012) surface to elucidate the origin of its activity and selectivity trends [13, 34]. During CO_2_RR on the catalyst surface, parallel reaction pathways were computationally evaluated, accounting for both CO_2_ reduction products and the competing HER [15, 16]. The reaction cascade initiates through a proton‐coupled electron transfer (PCET) process where CO_2_ activation occurs preferentially via C‐protonation rather than O‐protonation, as evidenced by DFT calculations. The formation of the *OCHO intermediate through C‐protonation represents the potential‐limiting step with a mildly endothermic energy barrier of +0.59 eV, while the subsequent PCET step leading to HCOO^−^ formation is exothermic (−0.28 eV). The weak adsorption energy of the formate product facilitates its spontaneous desorption from the catalyst surface. DFT calculations reveal striking differences in the thermodynamic feasibility of competing electrochemical processes on the Bi (012) surface (Figure 5b). Most notably, the O‐protonation pathway leading to *COOH formation—the critical intermediate for CO production—exhibits a substantially higher energy barrier (+1.27 eV) compared to the C‐protonation route (+0.59 eV), rendering this pathway energetically prohibitive under typical reaction conditions. Additionally, the free energy of H adsorption on the Bi (012) surface was too positive (+0.88 eV), preventing active HER. These fundamental insights explain the exceptional formate selectivity observed experimentally and highlight the unique catalytic properties of the Bi (012) surface in simultaneously suppressing both HER and CO production pathways while promoting selective CO_2_ reduction to formate.

After Sn doping, both the Bi sites (Sn@Bi_Bi_, Figure 5c) and Sn sites (Sn@Bi_Sn_, Figure S19) exhibit similarly low energy barriers for CO_2_ reduction to formate, mirroring the behavior of pristine Bi. This accounts for the experimental observation of formate as the dominant product. DFT calculations reveal that the improved FE for formate production originates from favorable changes in intermediate energetics induced by Sn doping. As shown in Figure 5d, the formation energy of *OCHO—the key intermediate governing HCOO^−^ production—decreases significantly at both the doped Bi and Sn sites compared to pristine Bi. This thermodynamic stabilization of *OCHO facilitates CO_2_ protonation while simultaneously suppressing competing pathways, accounting for the observed enhancement in formate selectivity. To further explore the influence of Sn doping on the electronic structures and bonding strength of adsorbates, Crystal Orbital Hamiltonian Population (COHP) analysis was conducted, and the integrated COHP (ICOHP) values for *OCHO adsorption through O were compared between pristine Bi(012) and Sn@Bi(012) surfaces (Figure 5e,f). As revealed by the COHP analysis in Figure 5e, the Bi‐OCHO interaction in the pristine crystal involves both bonding and antibonding orbital formations. Notably, the antibonding orbitals demonstrate a substantially greater ICOHP value compared to their bonding counterparts, indicating a net stabilizing interaction that facilitates *OCHO adsorption. Furthermore, after Sn doping, by comparing the COHP of Bi─O and Sn─O interactions, it is evident that the doped Sn induces stronger bonding in the neighboring Bi atoms. These findings are quantitatively supported by more negative ICOHP values and downshifted bonding peaks, both indicating stronger interactions [20, 38].

A comprehensive analysis of the projected density of states (PDOS) reveals that Sn doping induces significant electronic modifications in the Bi catalytic sites, profoundly influencing their interaction with key reaction intermediates. As demonstrated in Figure 5g, the p‐band center of Bi valence electrons undergoes a pronounced 0.26 eV upshift from 0.18 to 0.44 eV following Sn incorporation, indicative of substantial electron transfer from Bi to Sn atoms [20, 57]. The elevated p‐band center in doped Bi improves the energy alignment between Bi orbitals and the 2p orbitals of oxygen in *OCHO, thereby facilitating stronger orbital hybridization and superior binding. This electronic redistribution creates electron‐deficient Bi sites that exhibit enhanced stabilization of the electron‐rich *OCHO intermediate, as evidenced by charge density difference analysis (Figure S20). Comparative PDOS calculations of *OCHO adsorption configurations reveal that the p‐band center of Sn@Bi shifts significantly closer to the Fermi level relative to pristine Bi, enhancing orbital hybridization as evidenced by increased electron cloud overlap in Figure 5h. This metal‐oxygen interaction involves synergistic electron transfer mechanisms: σ‐donation from *OCHO lone pairs to unoccupied Bi orbitals, and p‐back‐donation from occupied Bi states to *OCHO antibonding orbitals. Charge density difference analysis (Figure 5j) provides direct visualization of these electronic effects, demonstrating markedly weaker *OCHO interaction with pristine Bi surfaces compared to doped configurations. The enhanced orbital mixing in Sn@Bi systems creates an optimal binding strength that stabilizes the critical *OCHO intermediate while maintaining sufficient lability for subsequent protonation steps, thereby explaining the improved formate production efficiency observed experimentally.

**FIGURE 6:**
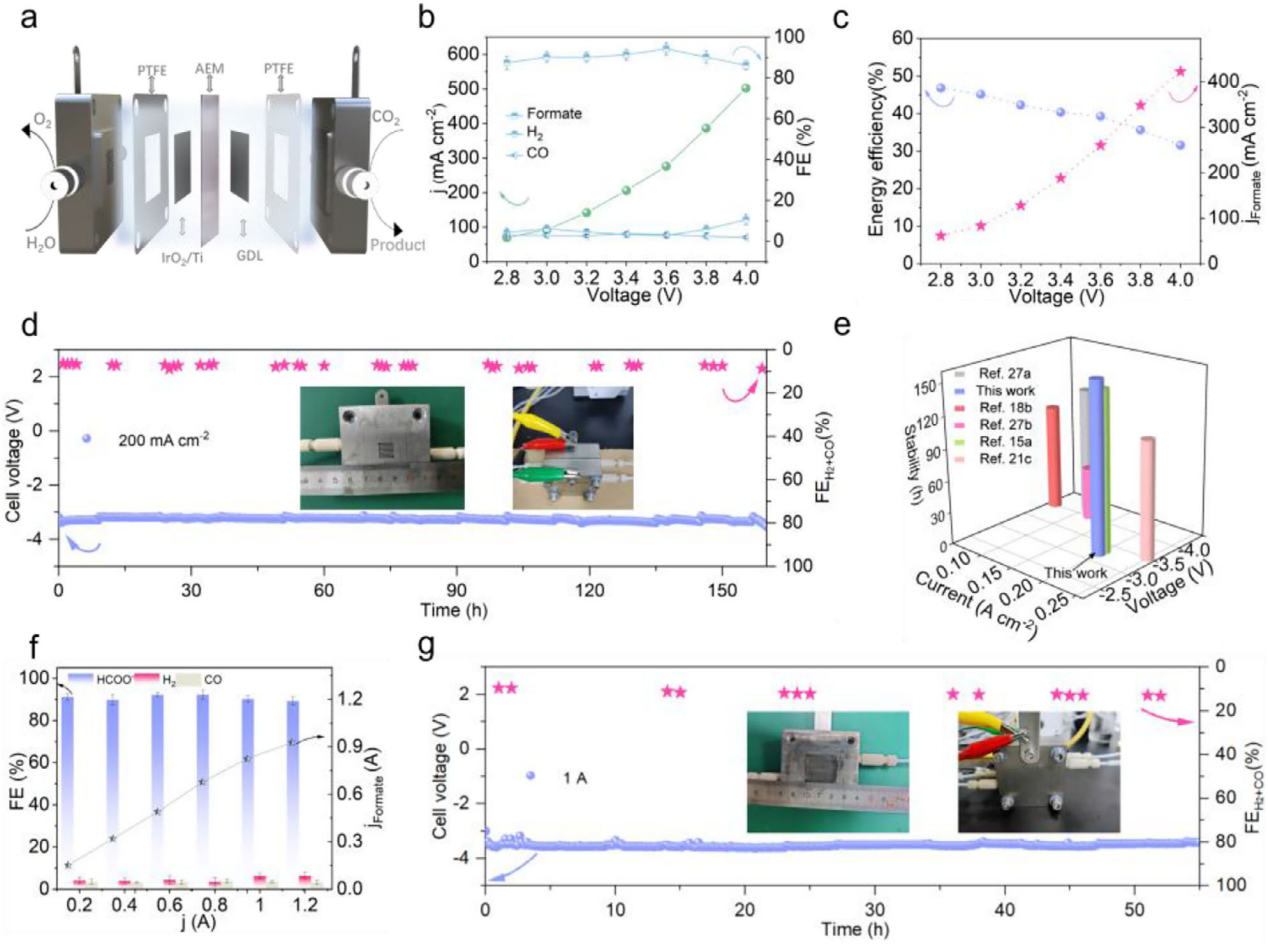
CO_2_RR performance of A‐BSO in MEA electrolyzer. (a) Schematic diagram of the two‐electrode MEA electrolyzer for CO_2_RR. (b) FE and current density at different cell voltages in 0.5 M KHCO_3_. (c) EE and partial current densities of formate at different cell voltages. (d) Stability test in a 1 cm^2^ MEA electrolyzer in 0.5 M KHCO_3_ at a current density of 200 mA cm^−2^. (e) Comparison of this work with advanced p‐block metal electrocatalysts in terms of current, voltage, and duration in MEA configuration. (f) FE and partial current densities of formate under different applied currents, and (g) continuous electrolysis under a constant total current of 1 A in a 4 cm^2^ MEA electrolyzer and corresponding FE_formate_ in 0.5 M KHCO_3_.

For practical applications, developing an MEA electrolyzer is crucial to advancing CO_2_RR technology. The sandwich‐structured MEA comprises a cathode, an anion exchange membrane, and an anode, with humidified CO_2_ gas fed to the cathode side (Figure 6a). First, an MEA electrolyzer with an active area of 1 cm^2^ was used to evaluate the performance of A‐BSO (Figure S21). The removal of catholyte substantially reduced the overall ohmic resistance of electrolyzer, thereby enhancing EE (Figure S22a). LSV tests revealed that, without iR compensation, the current density reached nearly 600 mA cm^−2^ as the polarization voltage increased to −4 V. At various constant voltages, the catalyst maintained a formate FE of approximately 90% over a wide voltage range, achieving a peak FE of 94.3% at 3.6 V. During MEA testing, the overall FE experienced a slight decrease compared to the flow cell configuration. This phenomenon may be attributed to the following reasons: first, some products may penetrate through the gas diffusion layer into the CO_2_ gas flow channel, thereby escaping collection; second, certain products may cross through the anion exchange membrane to the anode side, where they undergo re‐oxidation. EE, defined as the ratio of the chemical energy stored in the target product to the electrical energy input, is a key parameter for the practical implementation of CO_2_RR. As the voltage increased from 2.8 to 4.0 V, the EE for formate decreased from 47% to 32%, while the partial current density for formate reached 430 mA cm^−2^, indicating that formate production effectively utilized most of the electrical energy over a broad operating range (Figure 6c). Long‐term stability evaluation under industrial operating conditions confirms the exceptional durability of the A‐BSO catalyst, with continuous 160‐h testing in an MEA configuration demonstrating maintained cell voltage stability at 3.2 ± 0.2 V while suppressing competing reactions (FE_H2+CO_ < 10%) (Figure 6d). Compared to other recently reported p‐block metal‐based electrocatalysts, the A‐BSO demonstrated exceptional CO_2_RR performance, excelling in both activity and stability within the MEA system, and exhibited stable operation over extended periods under high‐current conditions (Figure 6e) [34, 41, 48, 58, 59].

To further validate the scalability potential of the A‐BSO catalyst in MEA systems, the CO_2_RR performance of a scaled‐up MEA with an active area of 4 cm^2^ was evaluated. The current‐voltage response of the A‐BSO electrocatalyst under a constant CO_2_ flow rate (25 sccm) is shown in Figure S23. As the current increased from 200 mA to 1.2 A, the selectivity for formate production remained consistently around 90%, with the partial current density for formate gradually increasing. Additionally, a 55‐h stability test at 1 A further confirmed the scalability of the electrochemical performance of A‐BSO. These results highlight the promising industrial applicability of the A‐BSO catalyst.

**FIGURE 7:**
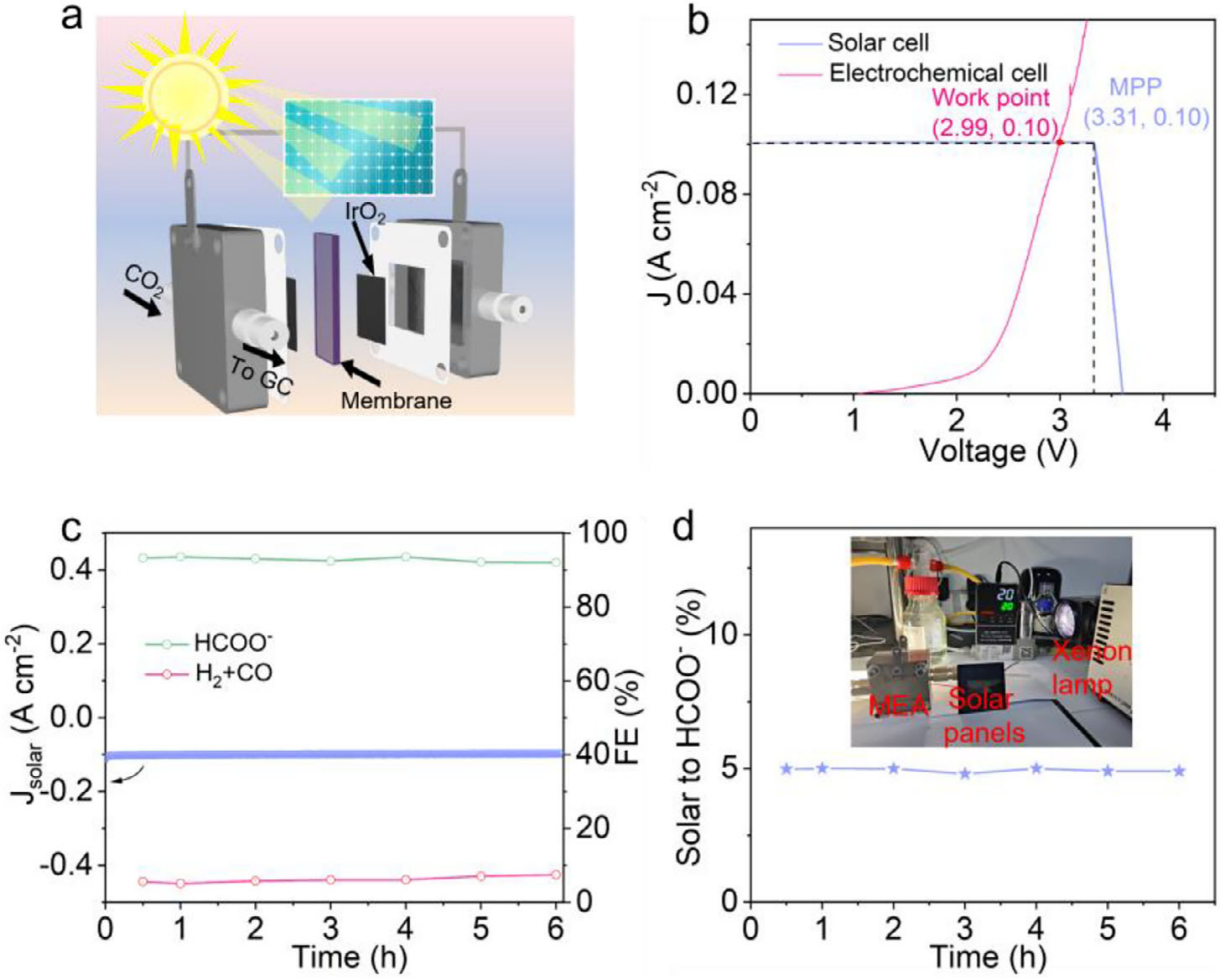
Solar‐driven overall CO_2_ splitting. (a) Schematic diagram of a solar‐driven CO_2_‐H_2_O overall splitting system. (b) I–V curve of a solar cell and an electrochemical cell. (c) Solar‐driven current density and corresponding FE. (d) Solar‐to‐formate conversion efficiency. The inset shows the complete solar‐powered electrolysis system.

Motivated by the outstanding CO_2_RR performance of A‐BSO, a CO_2_‐H_2_O overall splitting system in 0.5 M KHCO_3_ electrolyte was driven exclusively by a commercial monocrystalline silicon solar cell (5 × 5 cm^2^) as the sole energy source (Figure 7a) [32, 60]. When exposed to AM 1.5 G sunlight irradiation (100 mW cm^−2^), the solar cell demonstrated an open‐circuit voltage (V_oc_) of 3.63 V, a short‐circuit current (J_sc_) of 0.10 A, and a maximum power point (MPP) at 3.31 V (Figure S24). The system operating point was defined by the intersection of the electrochemical cell's polarization curve and the solar cell's current–voltage (I–V) curve, positioned near the MPP to ensure efficient solar energy utilization (Figure 7b). It should be noted that this is only an approximate value, as the resistance of the solar panel varies with temperature, and the response current of the MEA is not constant. The solar‐driven system achieved a current density of nearly 100 mA cm^−2^, with an average FE_HCOO_^−^ exceeding 90% throughout the splitting reaction process, demonstrating the high selectivity and stability of A‐BSO (Figure 7c). The overall solar‐to‐formate conversion efficiency was calculated to be approximately 5%, which is also limited by the conversion efficiency of the monocrystalline silicon solar cell (Figure 7d). The most straightforward approach to improving the conversion efficiency is to employ solar panels with higher photovoltaic efficiency and to optimize the power matching between the MEA and the solar panels. This innovative strategy of coupling electrocatalytic CO_2_ reduction with solar energy opens a new pathway for efficient and clean energy conversion.

## 3 Conclusions

In this study, we successfully synthesized an amorphous Bi‐based catalyst (A‐BSO) for stable CO_2_RR to formate. Experimental results revealed that, unlike its crystalline Bi_2_Sn_2_O_7_ counterpart, this amorphous catalyst undergoes fast reconstruction into Sn‐doped Bi (Sn@Bi) during the reduction process. It exhibits outstanding performance for formate production, achieving a FE of ∼95.6% at 800 mA cm^−2^ in a flow cell and sustaining a current density of 500 mA cm^−2^ in a MEA electrolyzer. Moreover, it demonstrates remarkable stability, maintaining an average FE of ∼92.3% over 160 h at 200 mA cm^−2^. Through in situ spectroscopic analysis and DFT calculations, we demonstrate that Sn doping modulates the electronic structure of Bi, enhancing the adsorption of the *OCHO intermediate, which is critical for formate generation. To enable renewable energy‐driven CO_2_‐to‐formate conversion, we powered the MEA electrolyzer using a monocrystalline silicon solar panel, successfully achieving stable operation at ∼100 mA cm^−2^ with an overall EE of ∼5%. This work provides valuable insights into the design of highly efficient and stable amorphous catalysts for CO_2_RR while demonstrating a practical approach to solar‐powered formate synthesis.

## Conflicts of Interest

The authors declare no conflicts of interest.

## Supporting information




**Supporting File**: advs73580‐sup‐0001‐SuppMat.docx.

## Data Availability

The data that support the findings of this study are available from the corresponding author upon reasonable request.;
