# Supplementary material for: Amorphous BiSnxOy for Efficient CO2 Electroreduction to Formate via In Situ Doping
Source: Adv Sci (Weinh). 2025 Dec 30;13(10):e22395. doi: 10.1002/advs.202522395 (PMC12915184; doi:10.1002/advs.202522395)
Supplement: Supplementary file 1 — Supporting File: advs73580‐sup‐0001‐SuppMat.docx. [file ADVS-13-e22395-s001.docx]

**Supporting Information**

**Amorphous BiSn_x_O_y_ for Efficient CO_2_ Electroreduction to Formate via In-Situ Doping**

*Zhenjie Cheng, Junnan Song, Lijia Liu,* *Chenglong Qiu, Lu Wang^,^* and Jiacheng Wang**

**Experimental Section**

**Chemicals**: Bismuth nitrate pentahydrate (Bi(NO_3_)_3_·5H_2_O, 99%), sodium stannate trihydrate (Na_2_SnO_3_·3H_2_O, 98%), sodium hydroxide (NaOH, 98%), tripotassium Citrate (C_6_H_5_K_3_O_7_, 98%), sodium formate (HCOONa, 99.5%), Tin(IV) oxide (SnO_2_, 99.9%), potasio hidroxido (KOH, 99%), potassium bicarbonate (KHCO_3_, 99.5%), dimethyl sulfoxide-d (DMSO-d6, 99.9%), Deuterium oxide (D_2_O, 99.9%), isopropanol (C_3_H_8_O, 99.7%), ethylene glycol, (C_2_H_6_O_2_, 99%), ethanol (CH_3_CH_2_OH, 99.5%) and iridium oxide (IrO_2_, 99.9%) were purchased from Aladdin. All the chemicals were used without further purification.

**Synthesis of C-BSO and A-BSO Nanoparticles:** Both crystalline (C-BSO) and amorphous (A-BSO) bismuth-tin oxide nanoparticles were synthesized via a hydrothermal method with slight modifications in precursor composition. For C-BSO, a mixture of 2.5 mmol Bi(NO_3_)_3_·5H_2_O and 3 mmol Na_2_SnO_3_·3H_2_O was dissolved in 20 mL of 1.25 M NaOH aqueous solution. For A-BSO, 2.5 mmol Bi(NO_3_)_3_·5H_2_O and 3 mmol SnO_2_ were dissolved in the same NaOH solution, followed by the addition of 2 mmol potassium citrate. After stirring at room temperature for 30 min, each solution was transferred into a Teflon-lined autoclave and heated at 200°C for 18 h. The resulting products were collected by filtration, washed three times with deionized water, and dried at 60°C for 24 h.

**Synthesis of Bi_2_O_3_ Nanoparticles:** Bismuth oxide nanoparticles were synthesized via a hydrothermal method. Briefly, 2 mmol of bismuth nitrate pentahydrate (Bi(NO_3_)_3_·5H_2_O) was dissolved in 20 mL of ethylene glycol under vigorous stirring at room temperature until a homogeneous solution was obtained. Subsequently, 10 mL of an aqueous sodium hydroxide solution (2 M) was added dropwise, leading to the immediate formation of a white bismuth hydroxide (Bi(OH)_3_) precipitate. The mixture was then transferred into a 50 mL Teflon-lined stainless-steel autoclave and subjected to hydrothermal treatment at 160°C for 12 h. After cooling naturally to room temperature, the resulting product was collected by centrifugation, washed repeatedly with ethanol and deionized water to remove residual impurities, and dried overnight at 60 °C.

**Characterization:** The crystalline phases of the catalysts were characterized by X-ray diffraction (XRD) using a Bruker D8 Advance diffractometer with Cu-Kα radiation (λ = 1.5406 Å). Morphological analysis and elemental composition were examined by field-emission scanning electron microscopy (FESEM, ZEISS Gemini 300) equipped with energy-dispersive X-ray spectroscopy (EDS, Bruker XFlash 61100). Surface chemical states were analyzed by X-ray photoelectron spectroscopy (XPS) on an ESCALAB 250 system using monochromatic Al Kα radiation (hν = 1486.6 eV). High-angle annular dark-field scanning transmission electron microscopy (HAADF-STEM) and elemental mapping were performed using a Thermo Scientific Talos F200i transmission electron microscope. Inductively coupled plasma mass spectrometry (ICP-MS) measurements were conducted on a Thermo Scientific iCAP RQ instrument. Electron paramagnetic resonance (EPR) spectra were recorded at room temperature using a JEOL JES-FA200 spectrometer operating at X-band frequency (∼9.4 GHz). X-ray absorption spectroscopy (XAS) measurements, including X-ray absorption near-edge structure (XANES) and extended X-ray absorption fine structure (EXAFS) at the Bi L3-edge, were performed at beamline BL07 of the Taiwan Light Source. All spectra were measured in transmission mode.

*In-situ* Raman spectroscopy was recorded on Raman spectrometer (HORIBA LabRAW HR Evolution) equipped with a green laser of 532 nm. Electrolyte was 0.5 M KHCO_3_ solution, GDE work as the working electrode, an Ag/AgCl electrode as the reference electrode, and a Pt wire as the counter electrode.

*In-situ* ATR-SEIRAS spectra were collected by an FT-IR spectrometer (Bruker Vertex 80) equipped with an MCT-A detector. Infrared reflection-absorption spectral mode was adopted, and a CaF_2_ slice was used as the window where infrared light going through. 50 µL of catalyst ink was drop onto the glassy carbon electrode (with a diameter in 5 mm) to serve as the working electrode. The Pt wire and Ag/AgCl electrode were used as the counter and reference electrodes, respectively. All spectrum was collected at a resolution of 4 cm^-1^, and each single-beam spectrum was an average of 64 scans.

**Electrochemical measurements**: The CO_2_RR performance of the as-prepared catalysts was implemented in a H-cell, flow cell, and an MEA, respectively. For the H-cell, the electrocatalyst powder inks were prepared using a mixture of 0.5 ml isopropanol, .0.5 ml ethanol, 0.05 ml Nafion solution, and 5 mg of the catalysts, followed by ultrasonication for 40 min. The 0.2 ml of the ink was uniformly loaded onto a carbon paper (YLS-30T) with a catalyst loading of 1 mg cm^-2^, which was used as the working electrode, while a Pt plate as the counter electrode and Ag/AgCl as the reference electrode. The working electrode and counter electrode were separated by a cation-exchange membrane (CEM, Nafion 117). A CO_2_ gas flow (typically 20 mL min^-1^) was fed to the cathode side by a gas mass flow meter (Beijing Sevenstar flow Co., LTD), 0.5 M KHCO_3_ aqueous solution as the electrolyte. For the flow cell system, the electrocatalyst inks were prepared using a mixture of 10 mg catalysts, 1 ml isopropanol, 1 ml ethanol, and 0.08 ml Nafion solution. The ink was sprayed onto the carbon paper (YLS-30T) to yield a catalyst loading of 1 mg cm^–2^. Iridium oxide loaded titanium mesh (IrO_x_-Ti) and Ag/AgCl electrode were employed as the counter electrode and reference electrode, respectively. The potential values were referenced to the RHE according to the formula E (RHE)=E (Ag/AgCl)+0.198+0.059×pH. All the potential was recorded without iR-correction. The working electrode and reference electrode were separated by an anionic exchange membrane (Fumasep FAB-PK-130). The MEA was constructed by the cathode, an anion exchange membrane (Sustainion X37-50 Grade RT), and an IrO_x_-Ti anode. When test performance, a humidified CO_2_ gas flow (25 mL min^-1^) was fed to the cathode side and an aqueous solution of 0.5 M KHCO_3_ was circulated around the anode side. The EIS tests were performed with an amplitude of 5 mV in a frequency range from 0.1 Hz to 100 kHz. LSV measurements were performed at a scan rate of 50 mV s^-1^. The ECSA analysis was indeed performed in 0.5 M KHCO_3_ electrolyte by measuring the double-layer capacitance (Cdl) from CV curves within the non-Faradaic potential region. The activated sample was tested after undergoing reduction at a current density of 30 mA cm^-2^ for 2 hours, followed by a 1-hour resting period to allow for complete dissipation of polarization.

**CO_2_RR Product Analysis:** The gas phase products of CO_2_RR were detected by an on-line gas chromatography (GC, GC9790Ⅱ, FULI INSTRUMENTS) using high-purity Ar as the carrier gas with a thermal conductivity detector (TCD) for detecting hydrogen and two flame ionization detector (FID) for detecting carbon monoxide and other hydrocarbons. The Faradaic efficiency of gaseous products can be calculated:

$FE\left( \% \right)=\frac{\left( \frac{v}{60s/min} \right)\times\left( \frac{x}{24000 {cm}^{3}/mol} \right)\times N\times F}{J}$×100% (1)

Where *v* is the CO_2_ flow rate, *x* is the measured concentration of gaseous products, *N* is number of electrons required for the special product, *F* is the Faraday constant (96500 C mol^-1^), and *J* is the total current.

The liquid products were quantified by 1H NMR (Bruker 400 MHz spectrometer) with water suppression, in which 500 µL of electrolyte was mixed with 100 µL of D_2_O containing 10 µL dimethyl sulphoxide (DMSO) as the internal standard. The concentration of HCOO^-^ was determined by the standard curve of HCOO^-^ to DMSO peak area ratio. The Faradaic efficiency of the HCOO^-^ can be calculated by the following equation:

$FE\left( \% \right)=2\times F\times\frac{n_{formate}}{Q}\times100\%$ (2)

where *n* is the measured amount of HCOO^-^ (mol), and *Q* is the recorded total charge during the operation.

The full-cell energy efficiency (EE) of the HCOO^-^ product was calculated using

$EE\left( \% \right)=\frac{{(1.23-E}_{formate})}{E}\times{FE}_{formate}\times100\%$ (3)

Where *E_formate_* is the theoretical equilibrium potential (-0.2 V vs. RHE) for the HCOO^-^ formation, and *E_cell_* is the full-cell voltage without iR correction.

**Solar-Driven CO_2_-H_2_O System:** The MEA was used to construct a solar-driven CO_2_-H_2_O system, and the A-BSO electrode and IrO_x_-Ti were used as the working electrode and the counter electrode, respectively. A commercial monocrystalline silicon solar cell (Working area: 5*5 cm^2^) was used as only input energy under a 300 W Xe lamp with an AM 1.5 G filter (CEL-HXF300-S). Under laboratory working conditions, the open-circuit voltage was measured to be 3.3 V and the short-circuit current was 100 mA based on the I-V curve measurements.

The solar-to-formate conversion efficiency can be defined as:

$${ST}_{formate}=\frac{{\triangle E_{formate}\times J\times FE}_{formate}}{P_{solar}}\times100\%$$

Where △*E_formate_* is the equilibrium potential for CO_2_ conversion to HCOOH, *FE_foramte_* is the Faradaic efficiency for HCOOH production, *J* is the operating current density of the work system, and *P_solar_* is the power of input sunlight density (100 mW cm^-2^).

**DFT calculations**: All calculations were performed using spin-polarized density functional theory (DFT) as implemented in the Vienna ab initio simulation package (VASP) ^[1,2]^. The exchange-correlation functional was treated within the generalized gradient approximation (GGA) using the Perdew-Burke-Ernzerhof (PBE) formulation, with a plane-wave cutoff energy of 500 eV ^[3]^. The Brillouin zone was sampled using a 3 × 3 × 1 Monkhorst-Pack k-point grid for structural optimization ^[4]^. Electron-ion interactions were described by the projector augmented wave (PAW) method ^[5]^. To eliminate spurious periodic interactions, a vacuum layer of 18 Å was introduced in the direction normal to the surface. The convergence criteria for structural optimizations were set as follows: maximum atomic force < 0.02 eV/Å and energy change < 1×10^-5^ eV between consecutive steps. Van der Waals interactions were accounted for using the DFT-D3 semiempirical correction with Grimme's scheme ^[6]^.

The Gibbs free energy change (ΔG) for each elemental step is defined as^[7,8]^:

$$\Delta G=\Delta E+\Delta ZPE-T\Delta S$$

where ΔE and ΔZPE are the adsorption energy based on density functional theory calculations and the zero-point energy correction, respectively. T and ΔS represent the temperature and the entropy change, respectively.

Ab initio molecular dynamics (AIMD) simulations were also performed by VASP, using gamma centred k-point sampling. The simulations were performed using a timestep of 1 fs in NpT ensemble with a Langevin thermostat. The DFT optimized structure was heated from 100 K to 1200 K during a time of 2.5 ps, and then the equilibrium simulation lasted for 20 ps to generate the amorphous structure.


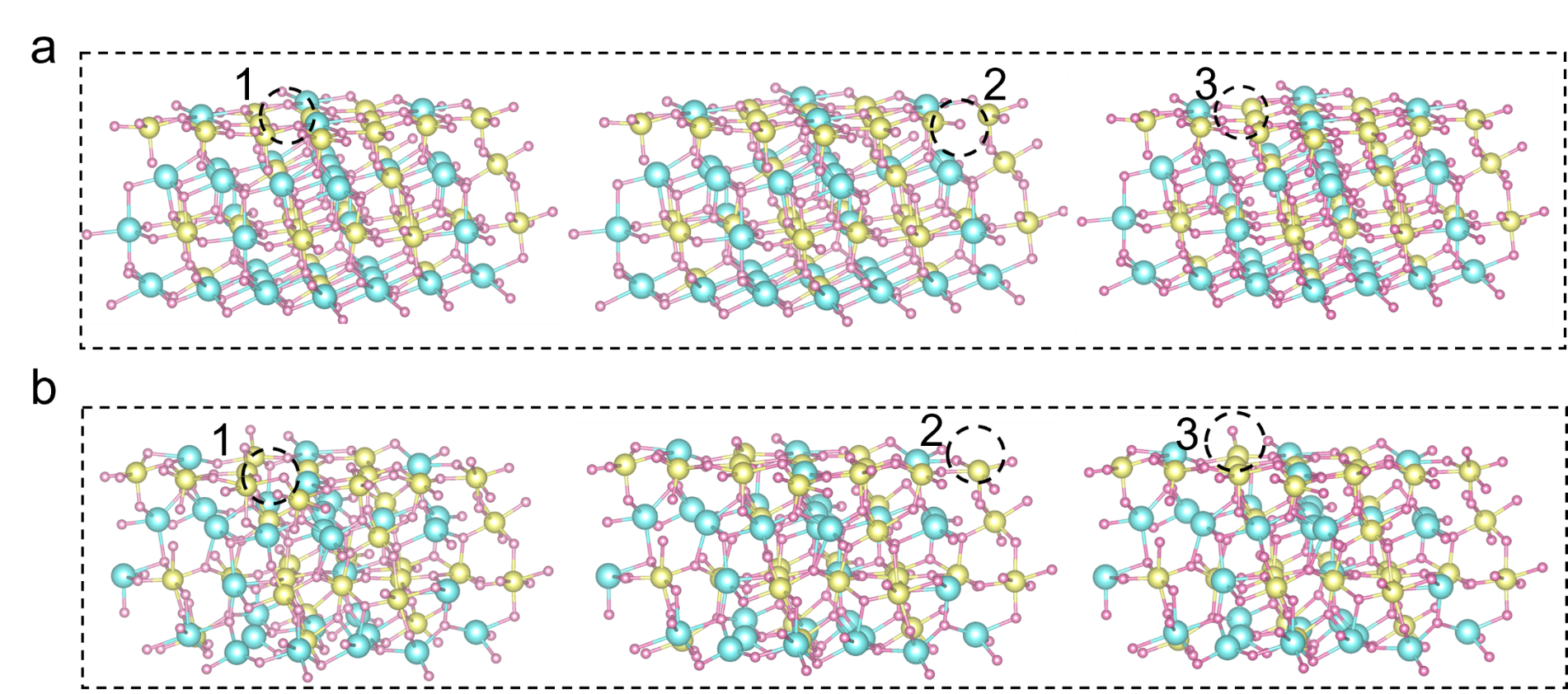


Figure S1**.** Schematic diagram of oxygen vacancies occurring at different sites in the C-BSO (a) and A-BSO (b).


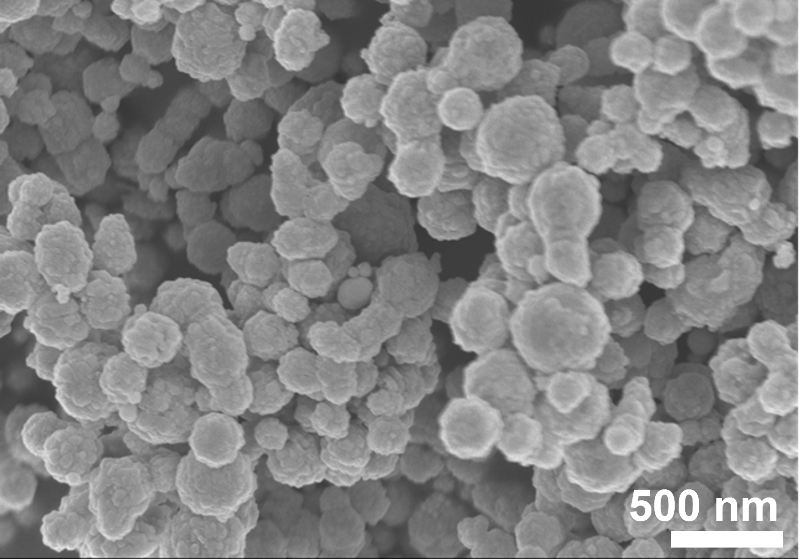


Figure S2. SEM image of pristine C-BSO catalyst.


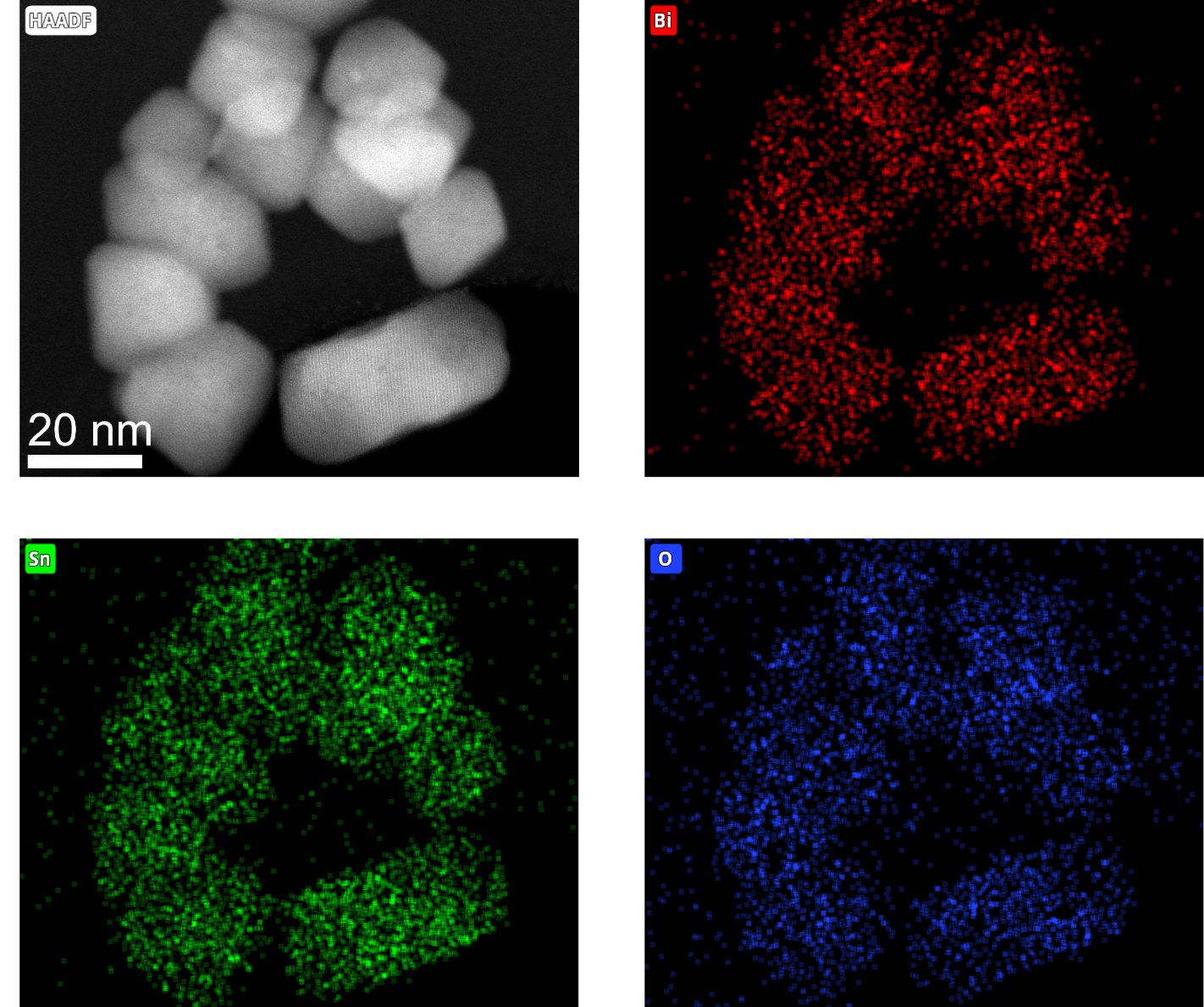


Figure S3. STEM image and EDS element mapping of Bi, Sn, and O for C-BSO sample.


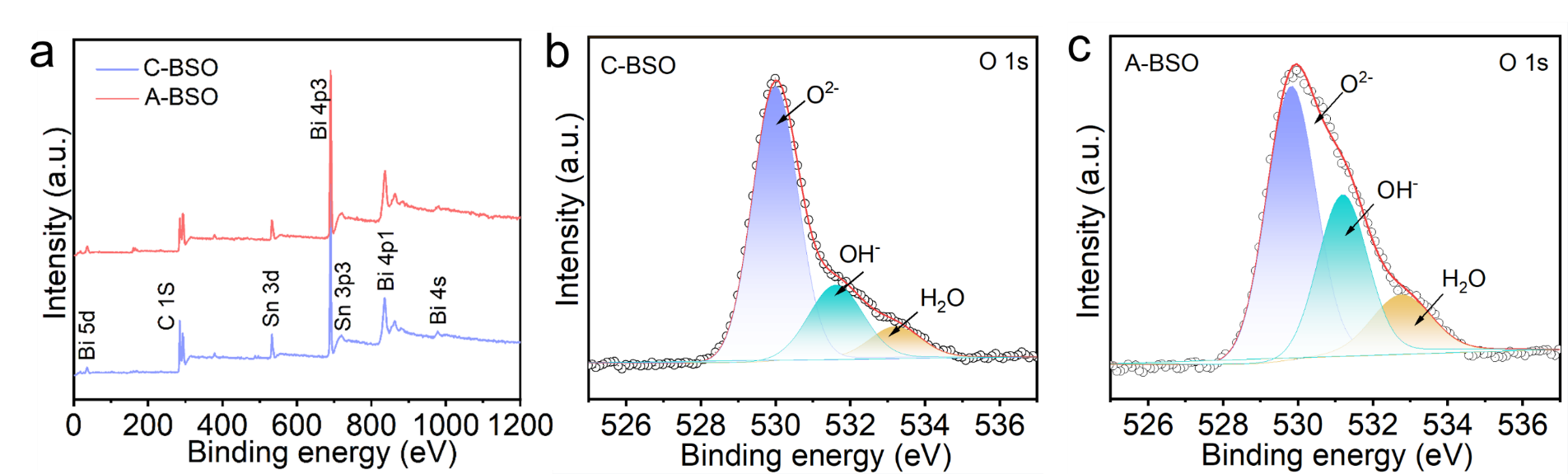


Figure S4. (a) XPS survey spectra of A-BSO and C-BSO. High-resolution XPS O 1s spectra for C-BSO (b) and A-BSO (c).


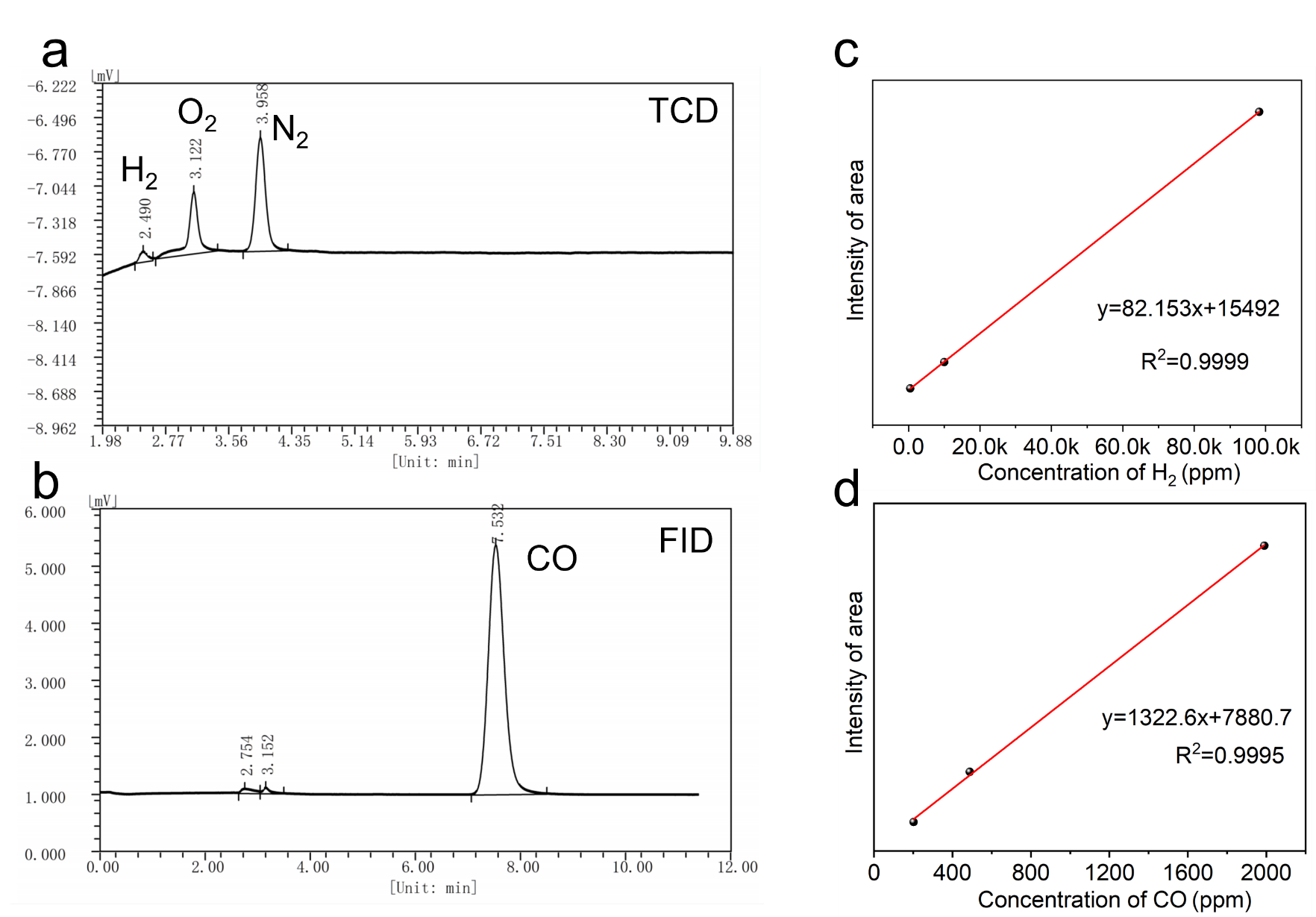


Figure S5. Quantification of H_2_ and CO concentration. Representative gas chromatography (GC) traces of H_2_ (a) and CO (b). Standard curves for H_2_ (c) and CO (d). The gas products were quantified by gas chromatography equipped with thermal conductivity detector (TCD) for analyzing H_2_ and a flame ionization detector (FID) for analyzing CO.


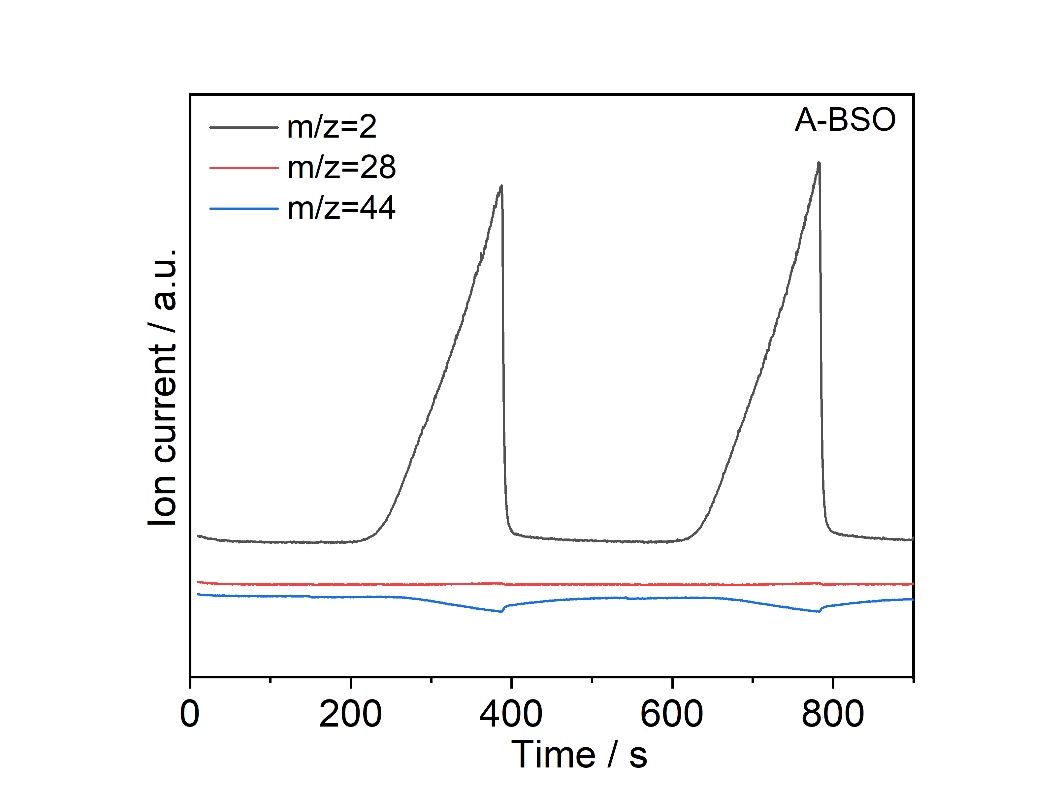


Figure S6. Ion current responses of m/z signal at different potentials.


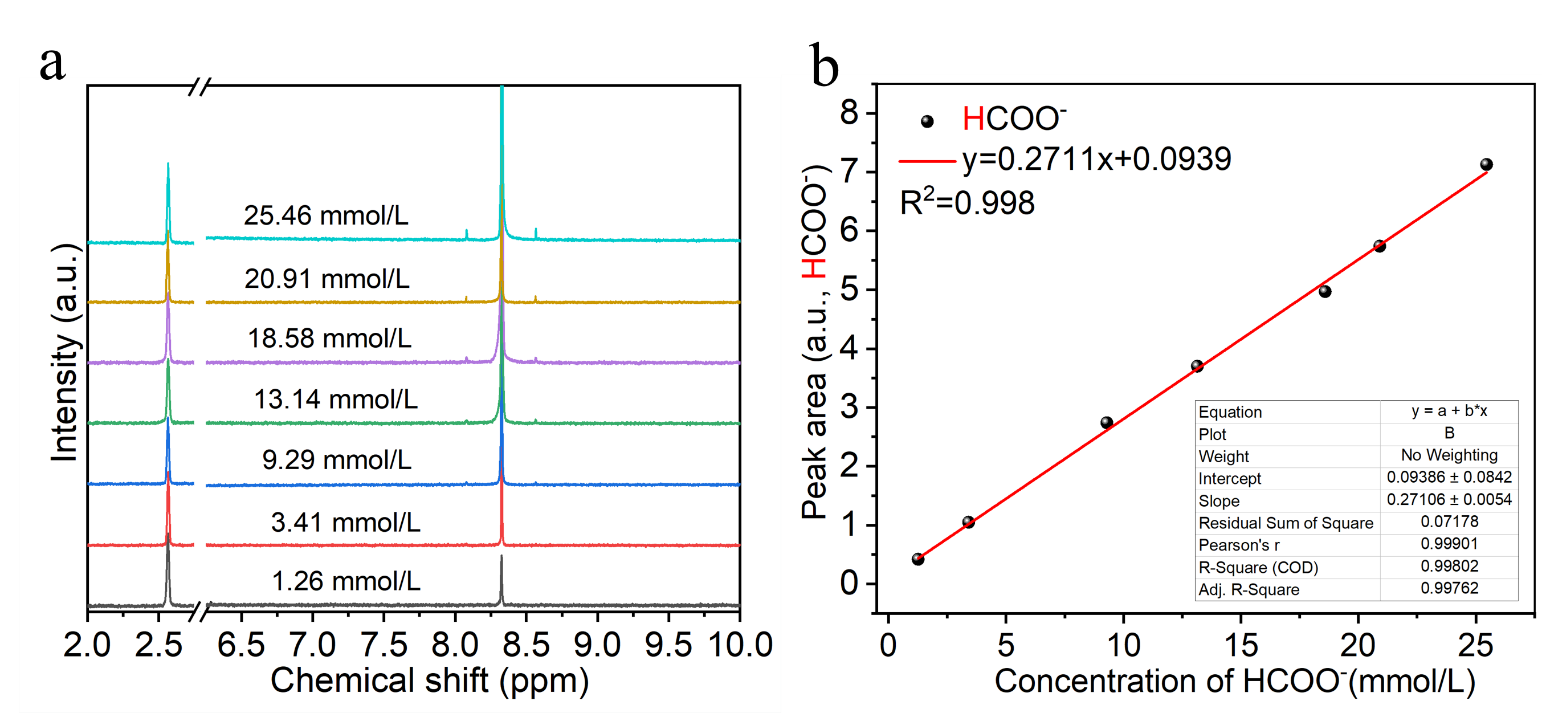


Figure S7. Quantification of HCOO^-^ concentration. (a) ^1^H NMR spectrum for HCOO^-^ in different concentrations. (b) Standard curve of HCOO^-^.


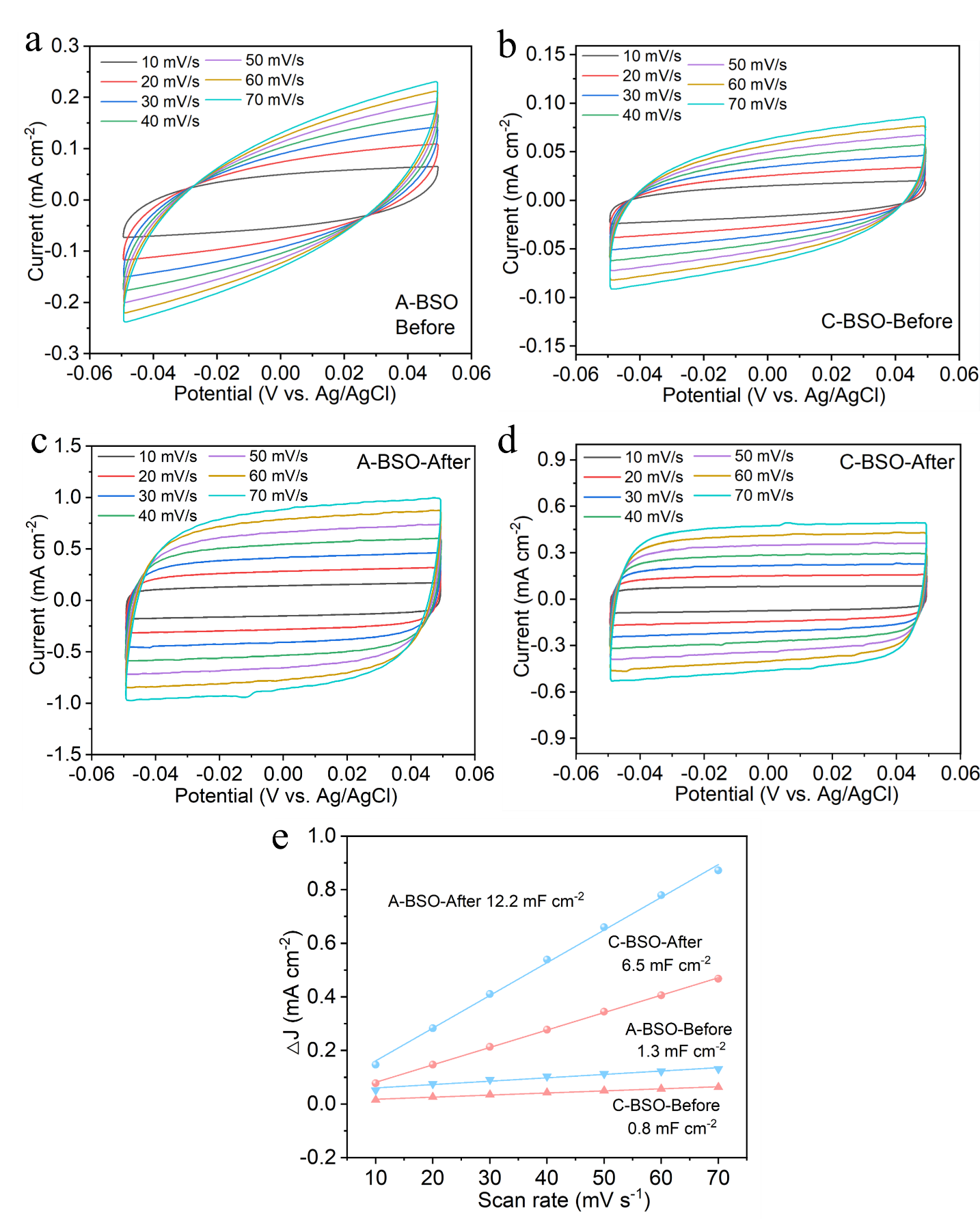


Figure S8. Evaluation of the specific electrochemically active surface areas. Cyclic voltammetry (CV) curves of the pristine materials: (a) A-BSO and (b) C-BSO, as well as those after activation: (c) A-BSO and (d) C-BSO. (e) The fitted slopes of the capacitive currents for these samples.


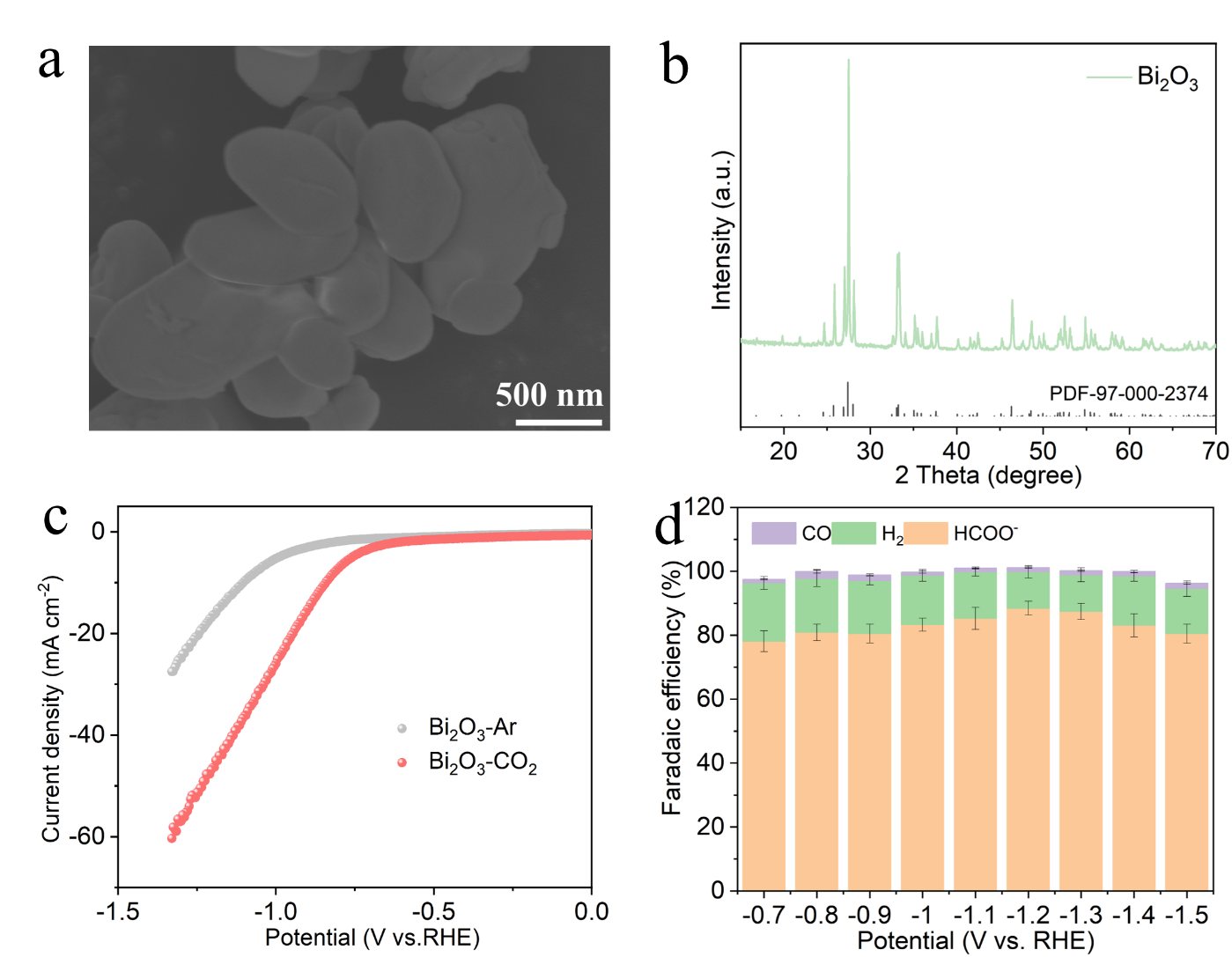


Figure S9. (a) SEM image and (b) XRD pattern of Bi_2_O_3_. (c) LSV curves and (e) FEs for HCOO⁻, CO, and H_2_ production in 0.5 M KHCO_3_ using Bi_2_O_3_ in an H-type cell.


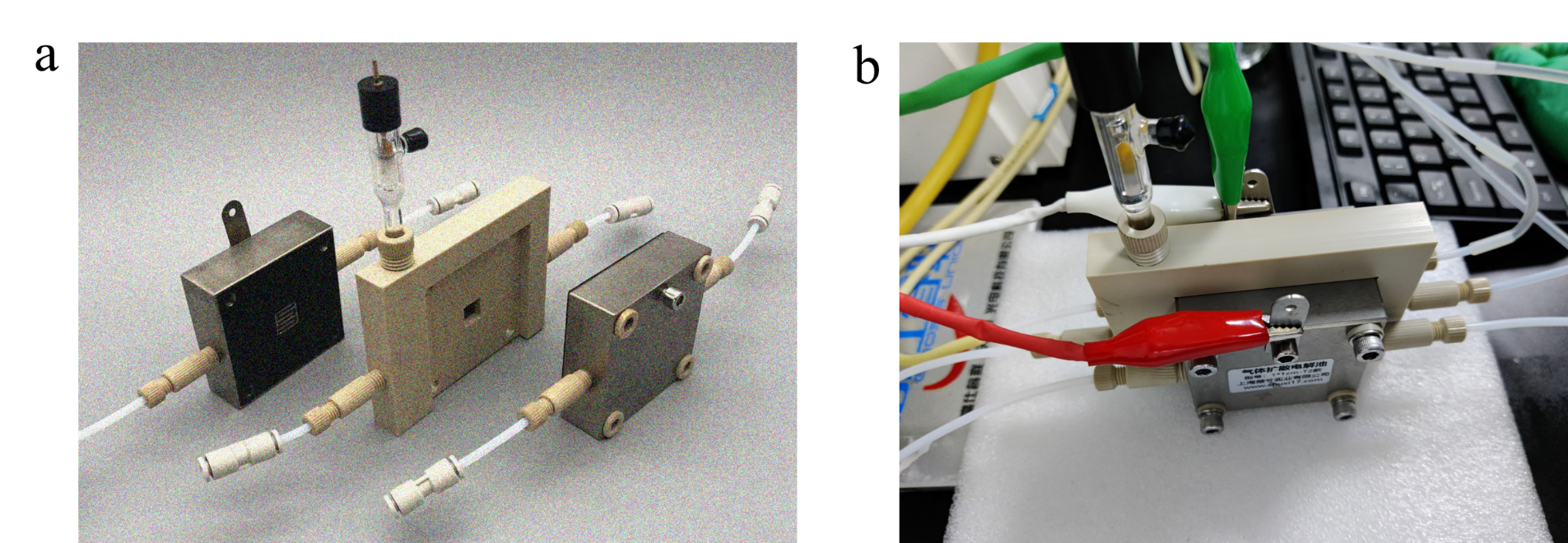


Figure S10. Optical image for alkaline flow cell electrolyzer.


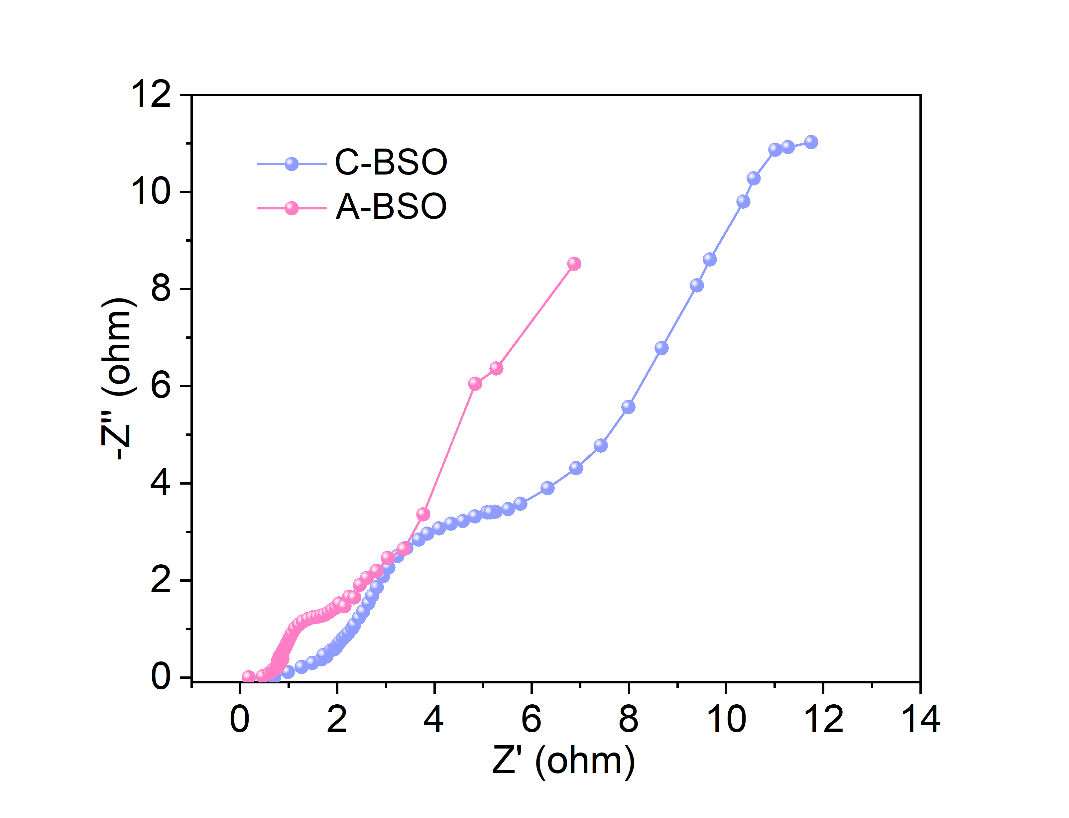


Figure S11. Electrochemical impedance plots of C-BSO and A-BSO electrodes.


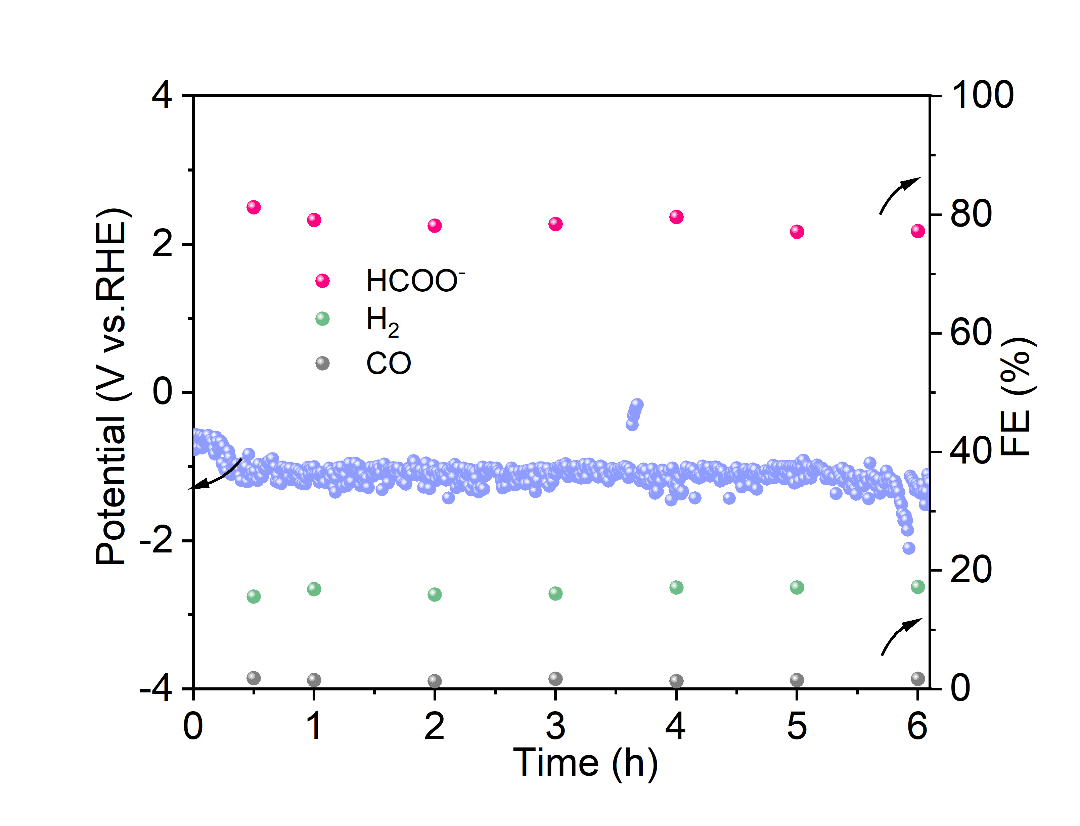


Figure S12. Stability testing at -200 mA cm^-2^ in 1 M KOH of C-BSO sample in the flow cell.


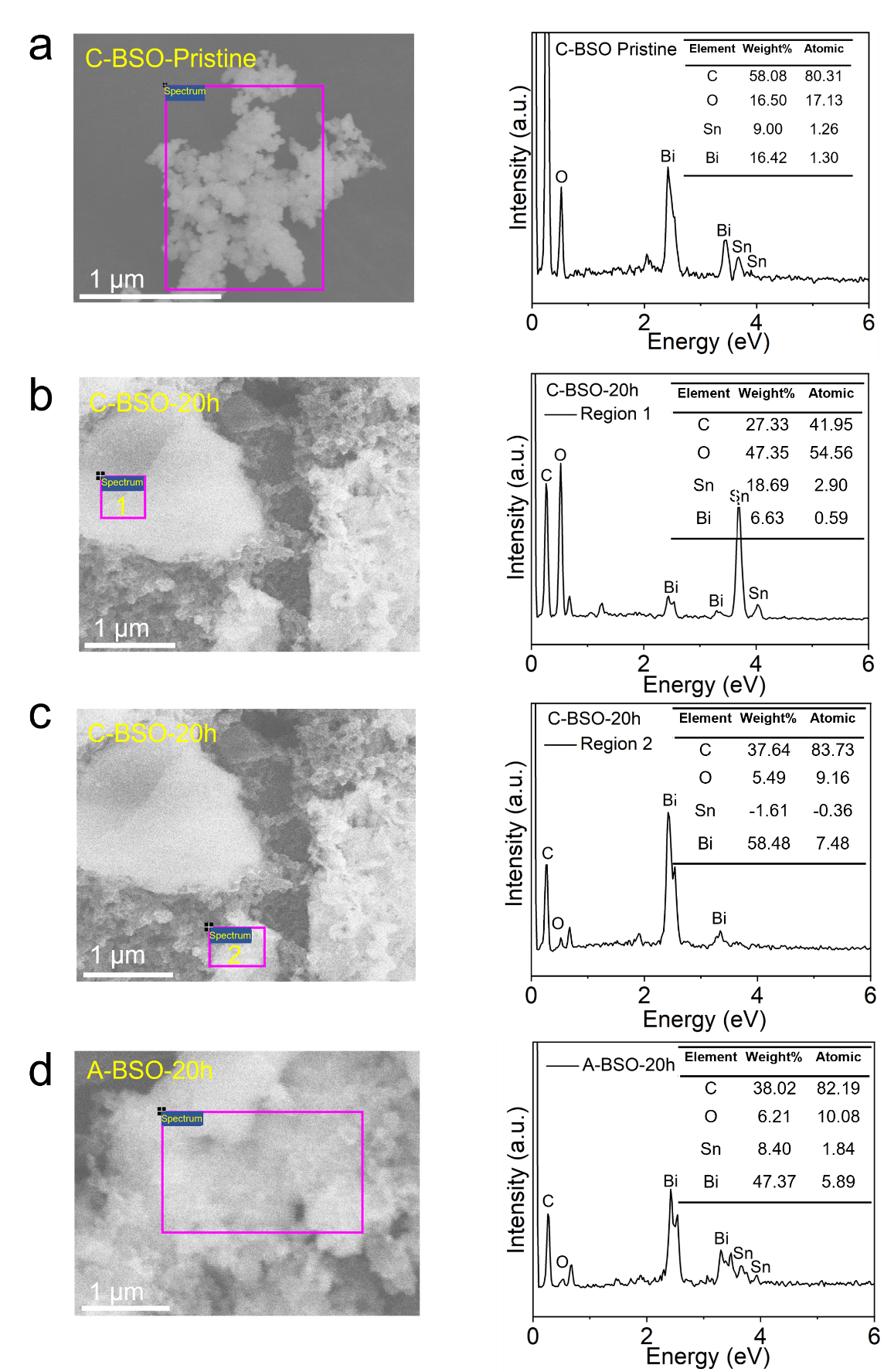


Figure S13. SEM images and corresponding EDS analysis of C-BSO and A-BSO samples: (a) pristine C-BSO; (b, c) C-BSO after 20 h of electrolysis; (e) A-BSO after 20 h of electrolysis.


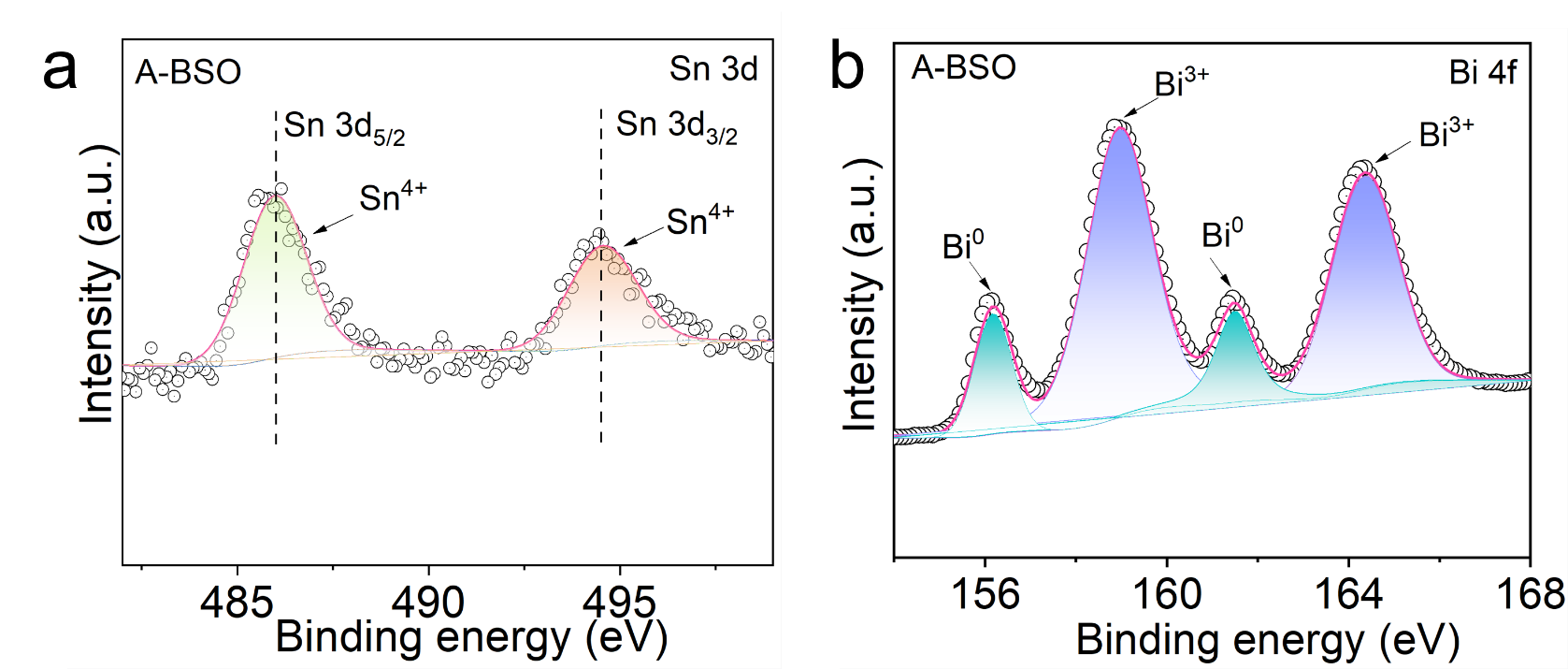


Figure S14. High-resolution (a) Sn 3d and (b) Bi 4f spectra for the reduced A-BSO


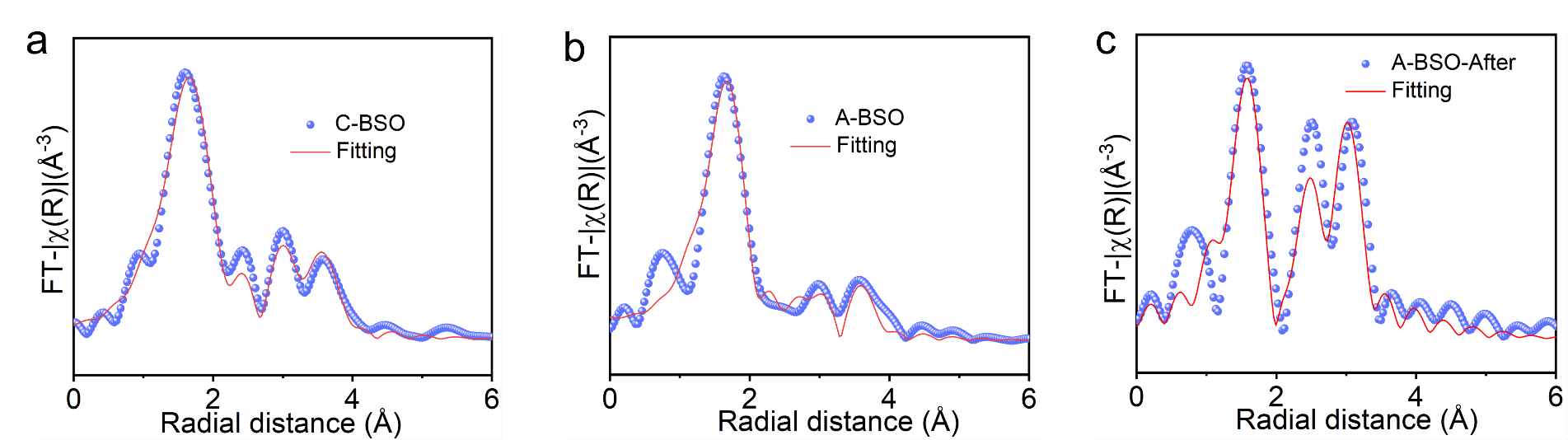


Figure S15. The Bi L_3_-edge EXAFS R space fitting curves for different samples.


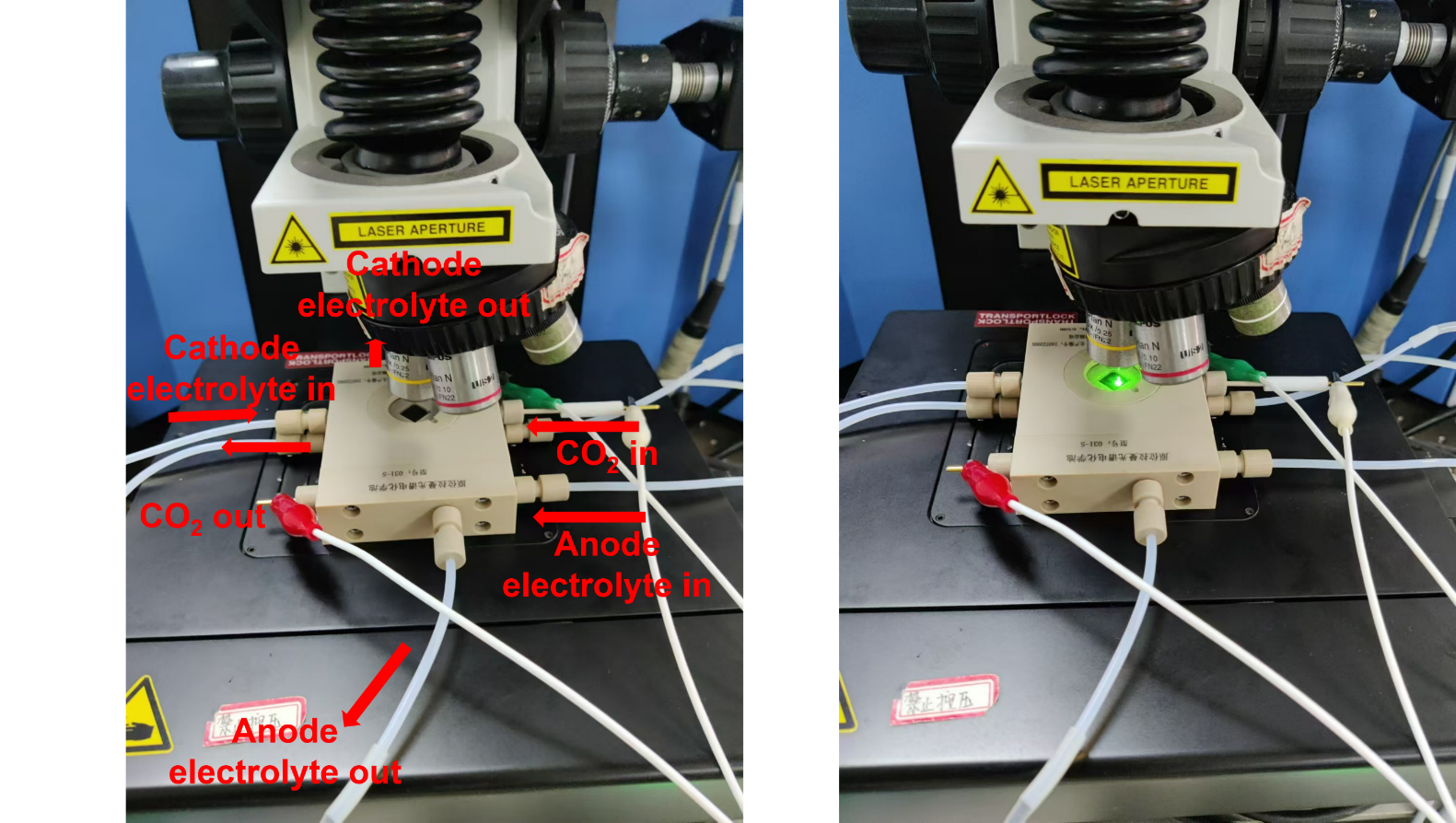


Figure S16. Optical photograph for in-situ Ramn measurements.


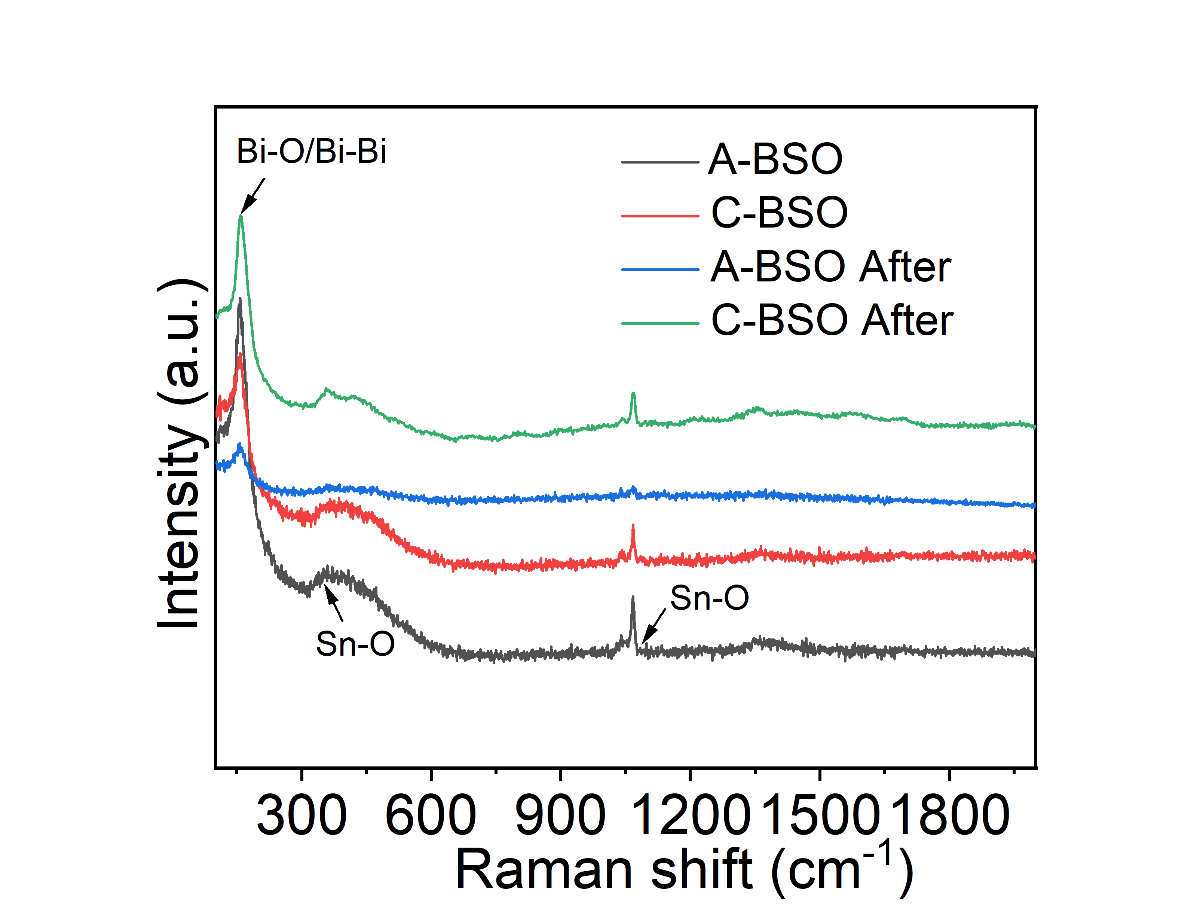


Figure S17. Ex-situ Raman spectra of different samples under varying conditions.


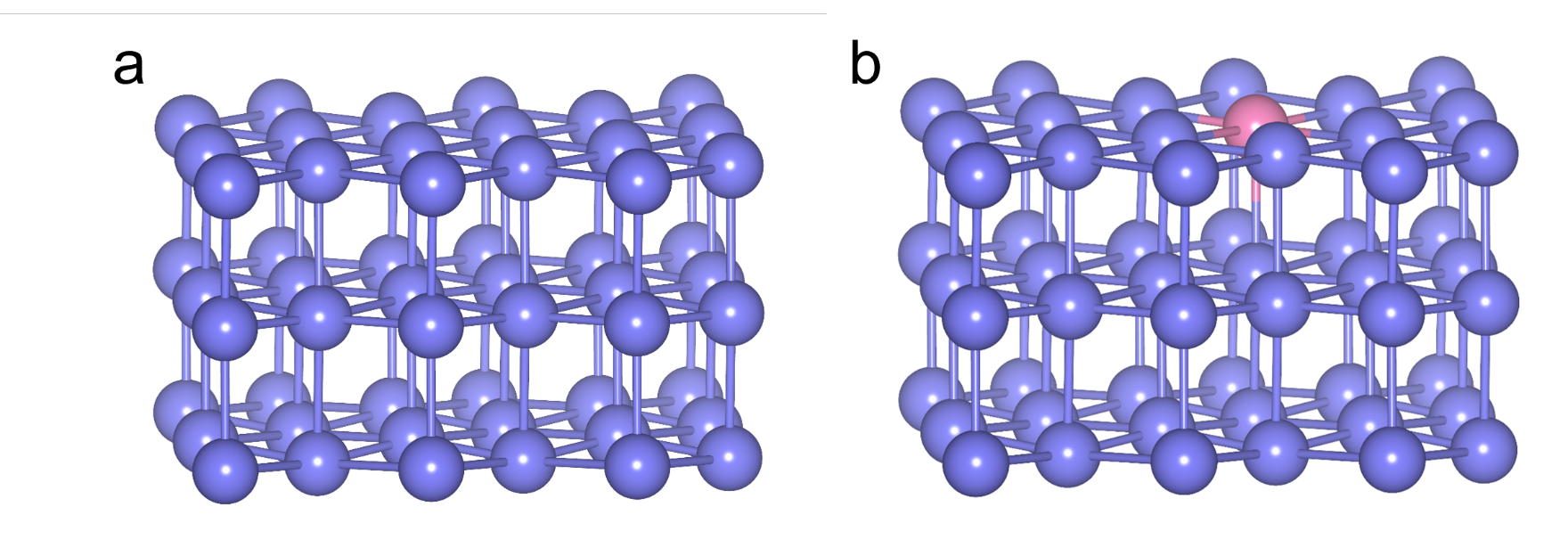


Figure S18 Optimized geometric structure of Bi (a) and Sn@Bi (b), where Bi and Sn atoms were presented by blue and pink spheres.


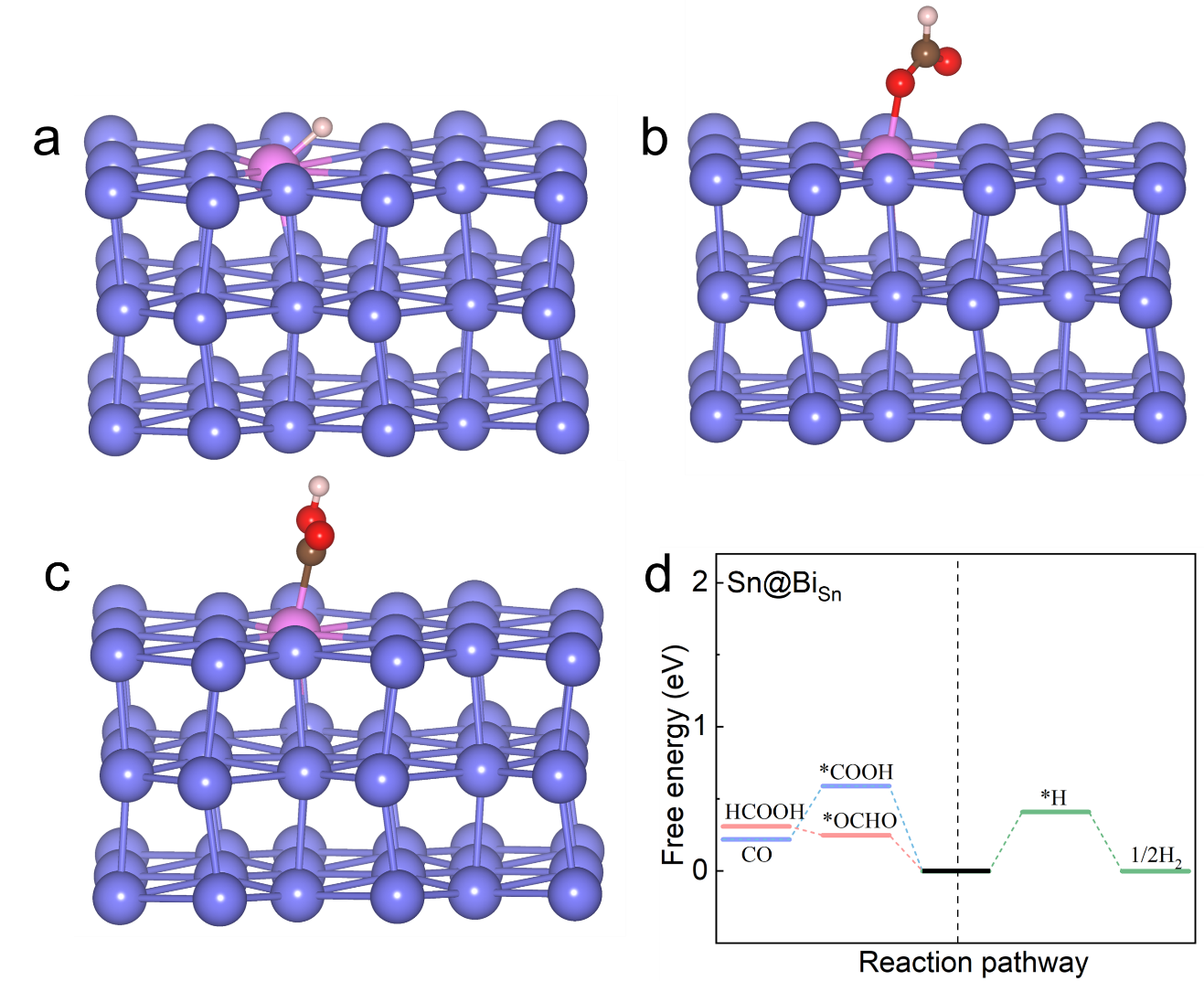


Figure S19 Optimized geometric structures of (a) H*, (b) *OCHO, and (c) *COOH adsorbates, as well as (d) the free energy diagrams of different adsorbates on the Sn@Bi (012) plane.


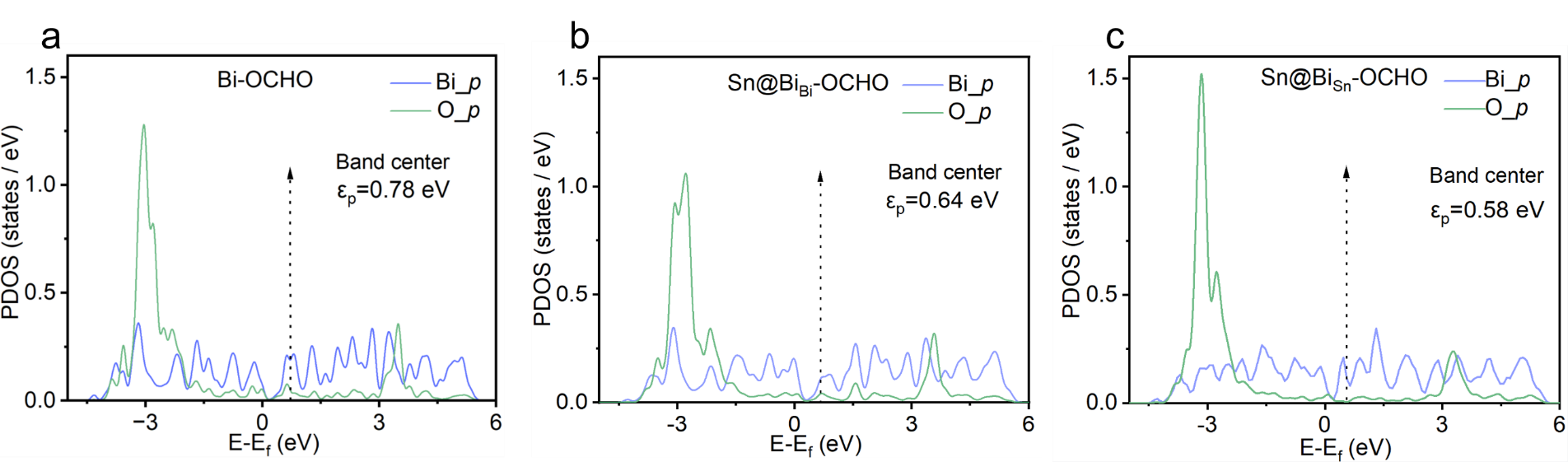


Figure S20 Projected p-orbital DOS of the Bi site with absorbed *OCHO.


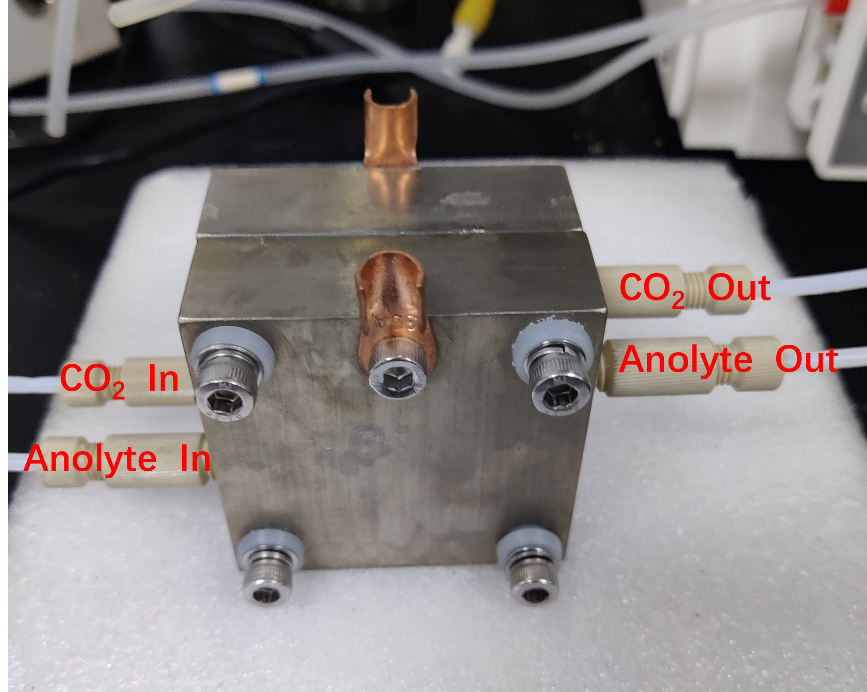


Figure S21 Optical image of 1 cm^2^ MEA electrolyzer.


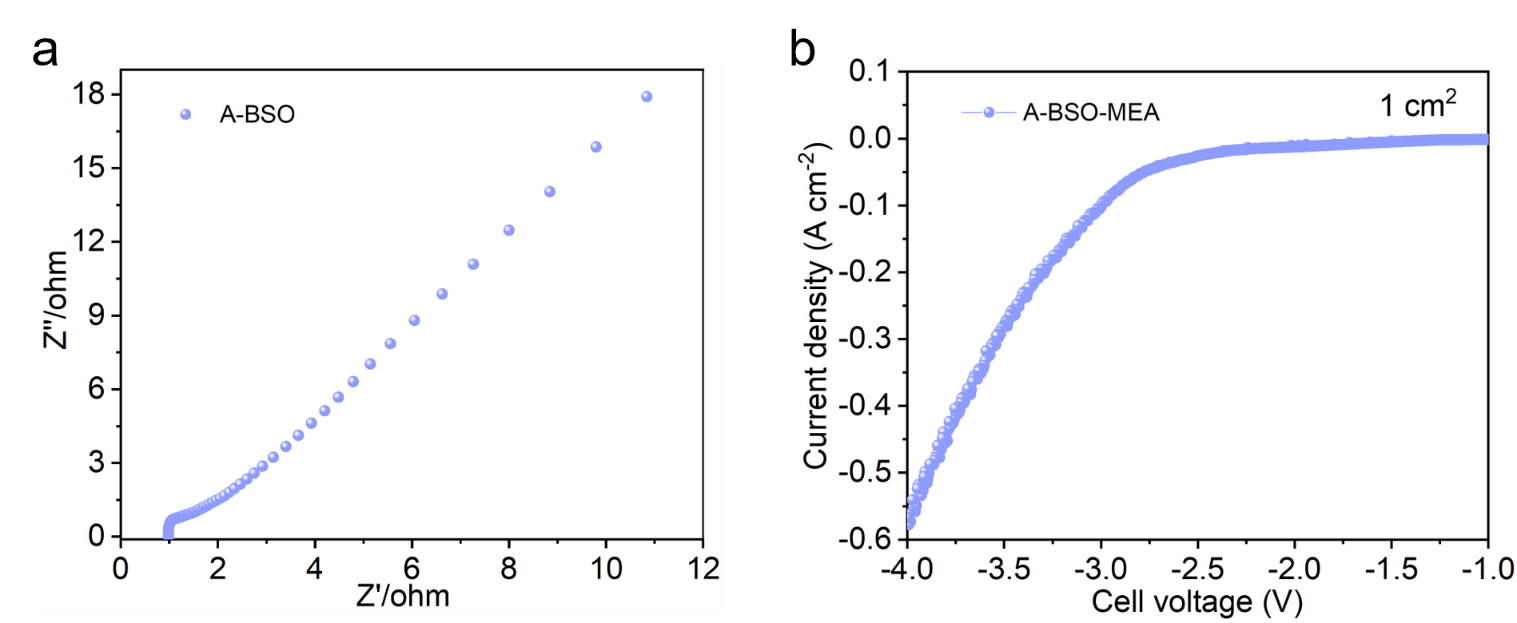


Figure S22 Electrochemical impedance plots (a) and LSV curve (b) of the MEA electrolyzer (1 cm^2^).


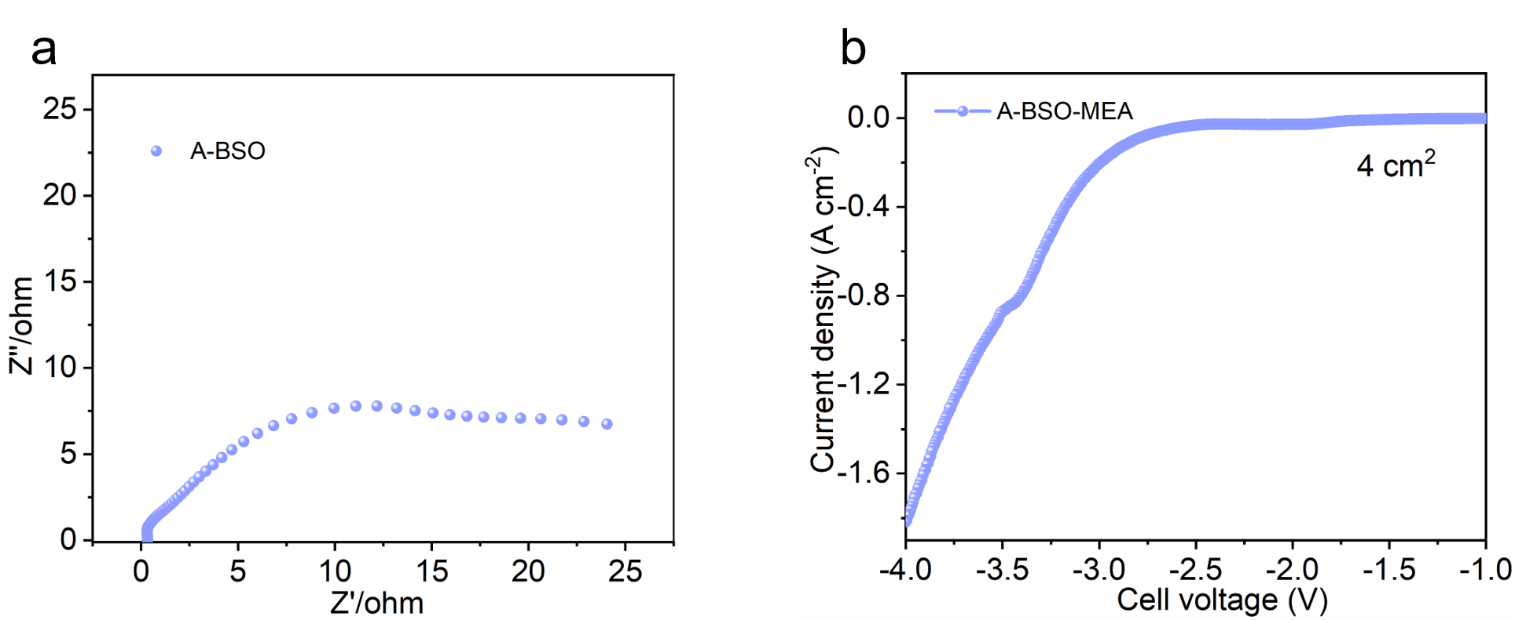


Figure S23 Electrochemical impedance plots (a) and LSV curve (b) of the MEA electrolyzer (4 cm^2^).


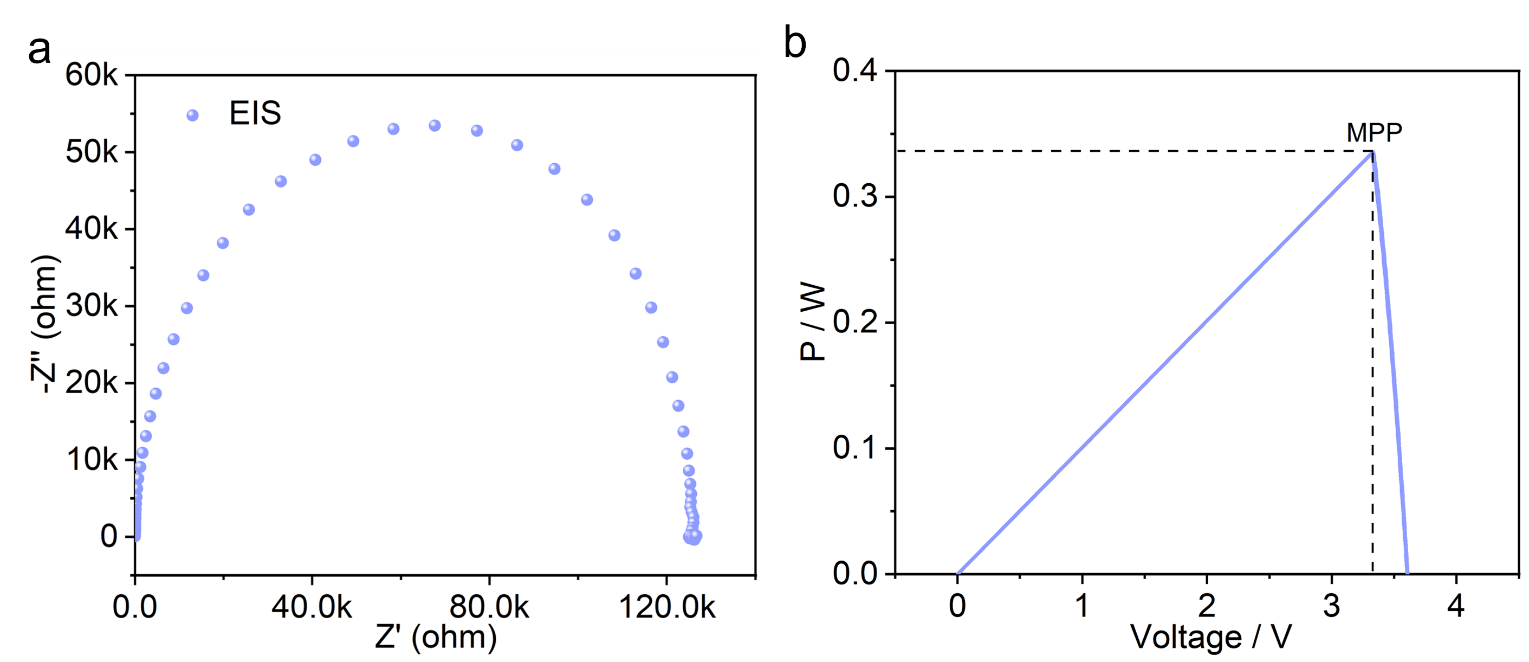


Figure S24 Electrochemical impedance plots (a) and output power curve (b) of Solar cell under AM 1.5 G sunlight (100 mW cm^-2^).

Table S1 The mass ratio of Bi to Sn in the A-BSO and C-BSO obtained by XPS and ICP-MS

| Bi:Sn (Mass Ratio) | C-BSO | A-BSO |
| --- | --- | --- |
| XPS | 1.70 | 5.86 |
| ICP | 1.81 | 6.13 |

**References:**

[1] G.Kresse, J. Furthmüller, Comput. Mater. Sci. 1 (1996) 15-50.

[2] G.Kresse, J. Furthmüller, Phys. Rev. B. 16 (1996) 11169-11186.

[3] J.P. Perdew, K. Burke, M. Ernzerhof, Phys. Rev. Lett. 18 (1996) 3865-3868.

[4] H.J. Monkhorst, J.D. Pack, Phys. Rev. B. 13 (12) (1976) 5188-5192.

[5] P.E. Blöchl, Phys. Rev. B. 50 (24) (1994) 17953-17979.

[6] S. Grimme, J. Antony, S. Ehrlich and H. Krieg, J .Chem. Phys. 132 (2010) 154104.

[7] J. Rossmeisl, A. Logadottir, J. K. Nørskov, Chem. Phys. 319 (2005) 178-184.

[8] A. A. Peterson, F. Abild-Pedersen, F. Studt, J. Rossmeisl, J. K. Nørskov, Energy Environ. Sci. 3 (2010) 1311-1315.
